# Tobacco control policies and perinatal health: a national quasi-experimental study

**DOI:** 10.1038/srep23907

**Published:** 2016-04-22

**Authors:** Myrthe J. Peelen, Aziz Sheikh, Marjolein Kok, Petra Hajenius, Luc J. Zimmermann, Boris W. Kramer, Chantal W. Hukkelhoven, Irwin K. Reiss, Ben W. Mol, Jasper V. Been

**Affiliations:** 1 Department of Obstetrics and Gynaecology, Academic Medical Center, Amsterdam, the Netherlands; 2 Centre for Medical Informatics, Usher Institute of Population Health Sciences and Informatics, The University of Edinburgh, Edinburgh, United Kingdom; 3 School for Public Health and Primary Care (CAPHRI), Maastricht University Medical Centre, Maastricht, the Netherlands; 4 Department of Paediatrics, Maastricht University Medical Centre, Maastricht, the Netherlands; 5 The Netherlands Perinatal Registry, Utrecht, the Netherlands; 6 Division of Neonatology, Erasmus University Medical Centre - Sophia Children’s Hospital, Rotterdam, the Netherlands; 7 The Robinson Institute, School of Pediatrics and Reproductive Health, University of Adelaide, Adelaide, Australia

## Abstract

We investigated whether changes in perinatal outcomes occurred following introduction of key tobacco control policies in the Netherlands: smoke-free legislation in workplaces plus a tobacco tax increase and mass media campaign (January-February 2004); and extension of the smoke-free law to the hospitality industry, accompanied by another tax increase and mass media campaign (July 2008). This was a national quasi-experimental study using Netherlands Perinatal Registry data (2000–2011; registration: ClinicalTrials.gov NCT02189265). Primary outcome measures were: perinatal mortality, preterm birth, and being small-for-gestational age (SGA). The association with timing of the tobacco control policies was investigated using interrupted time series logistic regression analyses with adjustment for confounders. Among 2,069,695 singleton births, there were 13,027 (0.6%) perinatal deaths, 116,043 (5.6%) preterm live-births and 187,966 (9.1%) SGA live-births. The 2004 policies were not associated with significant changes in the odds of developing any of the primary outcomes. After the 2008 policy change, a -4.4% (95% CI -2.4; -6.4, p < 0.001) decrease in odds of being SGA was observed. A reduction in SGA births, but not preterm birth or perinatal mortality, was observed in the Netherlands after extension of the smoke-free workplace law to bars and restaurants in conjunction with a tax increase and mass media campaign.

Tobacco use is associated with six million deaths annually making it the leading global cause of preventable death^1^. In addition, it is estimated that second-hand smoke (SHS) exposure causes 600,000 deaths worldwide each year^2^. The World Health Organization (WHO) recommends enforcement of 100% smoke-free environments through national legislation to reduce SHS^1^. Despite this effort, only an estimated 18% of the world’s population was covered by comprehensive smoke-free legislation in 2014^1^.

Compelling evidence supports a causal role of SHS exposure in the development of numerous health hazards in adults^3^, and the associated risks for infants and children are equally pertinent^2^. Already before birth, through maternal smoking and smoke exposure, the fetus is at risk for intrauterine death^4^, preterm birth^5^,^6^, and intrauterine growth restriction^6^.

Through reducing maternal smoking and SHS exposure, tobacco control policies have considerable potential to benefit perinatal health. Tobacco tax increases have been associated with dose-dependent reductions in key adverse perinatal and infant outcomes^7^,^8^,^9^,^10^. Studies evaluating regional or national laws prohibiting smoking in public places have shown important reductions in rates of stillbirth,^11^ neonatal mortality^11^, preterm birth^12^,^13^,^14^,^15^,^16^, low birth weight^11^,^13^,^15^, and small-for-gestational age (SGA) newborns^13^,^15^,^17^. Whereas a meta-analysis of adult studies showed a ‘dose-response’ association between comprehensiveness of smoke-free laws and their health impact^18^, whether such a ‘dose-dependent’ effect also applies to the impact of smoke-free legislation on early-life health is currently unclear^16^. To further assess this and accordingly inform policy makers on the relevance of tobacco control policies being comprehensive, additional studies are needed in countries that vary in this regard.

In the Netherlands there have been two key moments at which tobacco control policies have been jointly implemented. A smoke-free workplace law was introduced on January 1^st^, 2004, and was accompanied by a tobacco tax increase and mass media campaign. On July 1^st^, 2008 the smoke-free law was extended to the hospitality industry (i.e. bars and restaurants and sports, art and cultural venues), and at the same time another tobacco tax increase and mass media campaign were introduced. In this study we investigated whether these joint introductions of tobacco control policies in the Netherlands were associated with changes in key perinatal outcomes known to be associated with maternal smoking and/or SHS exposure.

## Methods

We undertook a national quasi-experimental study, which involved analysing the association between the 2004 and 2008 joint introduction of tobacco control policies and perinatal mortality, preterm birth, and SGA births using monthly data from a comprehensive dataset of singleton births in the Netherlands between 2000 and 2011. We pre-specified all analyses in a registered study protocol: ClinicalTrials.gov NCT02189265.

### Conceptual framework

**Figure 1:**
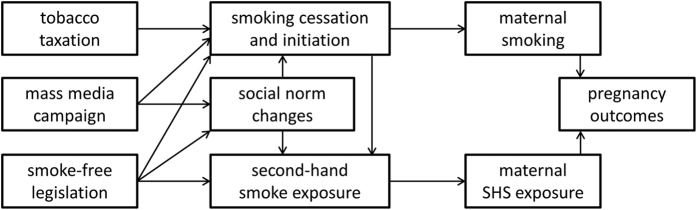
Directed acyclic graph of recognised pathways contributing to the link between tobacco control policies and pregnancy outcomes. Maternal smoking and second-hand smoke (SHS) exposure during pregnancy are associated with adverse pregnancy outcomes. Tobacco taxation has a direct impact on smoking rates, including maternal smoking during pregnancy. Mass media campaigns also have the potential to decrease maternal smoking and induce social norm changes which in turn affect smoking and SHS exposure. Smoke-free legislation reduces SHS exposure and is also associated with reduced smoking rates, both of which are in part mediated via social norm changes. Decreased smoking prevalence in itself also reduces SHS exposure. Tobacco taxation and smoke-free legislation have previously been associated with important reductions in adverse perinatal outcomes in multiple studies, supporting this conceptual framework.

Based on existing literature we have developed a conceptual framework underpinning this work, outlining the various ways in which tobacco control policies are likely to affect pregnancy outcomes (Fig. 1). Our dataset lacked reliable information on maternal smoking during pregnancy (see ‘Sensitivity analyses’).

### Data source

Individual-level health care data were obtained from the Netherlands Perinatal Registry (PRN) between 2000 and 2011, inclusive. The PRN database is a validated linkage of three different national registries: a midwifery registry (LVR1), an obstetrics registry (LVR2) and a neonatal registry (LNR)^19^. The PRN registry contains prospectively obtained population-based data on pregnancies, maternal and neonatal medical diagnoses, and provided care (interventions, referrals, deliveries and admissions) of newborns of approximately 96% of all deliveries in the Netherlands, including homebirths^19^.

### Inclusion and exclusion criteria

The study population comprised all registered singleton births (i.e. stillbirths and live-births) occurring between 2000 and 2011 between 24^+0^ to 42^+6^ weeks of gestation. Pregnancy dating was performed by last menstrual period and/or first trimester crown-rump length measurement using ultrasound. While the latter is known to have been used increasingly over the study period^20^, the exact method used was not recorded for individual cases in PRN. We excluded multiple pregnancies from the analysis because of their inherent increased risk for adverse pregnancy outcomes. Pregnancies of unknown gestation length, newborns weighing less than 500 grams at birth and newborns with recognised chromosomal anomalies were also excluded.

### Interventions

In 1990, the first smoke-free law was introduced in the Netherlands^21^. This ban made it illegal to smoke inside government agencies, healthcare facilities, educational institutions, social service facilities, and community work and indoor sports facilities. Compliance with this smoking ban was low and non-compliance was not adequately addressed (i.e. only through written warnings). In 2002, 36% of inspected educational institutions and 62% of government agencies were non-compliant^22^.

On January 1^st^, 2004 an amendment was introduced prohibiting smoking in workplaces and public transport, accompanied by a mass media campaign. Exceptions to this smoking ban were: hotels, bars and restaurants, sports, arts and culture venues, amusement arcades, tobacconist shops, international passenger transport, private spaces, open air, and designated areas for smoking within each facility^23^. Governmental inspections undertaken between six and 12 months after introduction of the smoke-free workplace law demonstrated violations of one or more aspects of the law in 32% of cases^24^. One month after introduction of the smoke-free law an excise tax increase was additionally introduced; cigarette prices increased by 19%^25^.

On July 1^st^, 2008, the smoke-free legislation was extended to include hospitality venues, making it illegal to smoke inside hotels, bars and restaurants, sports, art and culture venues, amusement arcades, tobacconist shops, and international passenger transport^26^. As with the smoke-free workplace law, designated indoor smoking areas were however still allowed. Introduction of the law was accompanied by a mass media smoking cessation campaign, as well as an 8% tax increase on tobacco products^27^. The legislation encountered much resistance, especially from bar owners. In several bars, the smoking ban was trespassed intentionally and fines were successfully challenged in court^28^.

On November 3^rd^, 2010 an exemption to the July 2008 smoking ban was made for bars with a surface area of 70 square meters or less, and with no other personnel than the owner^29^. On governmental inspection in winter 2008/2009, people were seen to be smoking in 6% of venues, including 26% of cafes, with significant gradual increases to 8% and 41%, respectively, in spring 2010^30^. A timeline indicating timing of these key tobacco control policies is presented as Supplemental Figure S1.

### Outcome measures

Our main outcome measures were: perinatal mortality (defined as all stillbirths and cases of early neonatal mortality (i.e. within the first seven days after birth) combined), preterm birth (live-birth before 37^+0^ weeks of gestation), and being born SGA (live-birth with birth weight below the 10^th^ centile). Secondary outcome measures were: stillbirth, early neonatal mortality, very preterm birth (live-birth before 32^+0^ weeks of gestation), low birth weight (live-birth with birth weight below 2,500 grams), very low birth weight (live-birth with birth weight below 1,500 grams), very SGA (live-birth with birth weight below 2.3^rd^ centile), and congenital anomalies. Congenital anomalies associated with antenatal smoke exposure were selected based on a systematic review on the association between maternal smoking in pregnancy and birth defects^31^. These were: cardiovascular/heart defects, musculoskeletal defects, missing/extra digits, limb reduction defects, clubfoot, craniosynostosis, facial defects, eye defects, orofacial clefts, gastrointestinal defects, gastroschizis, anal atresia, and abdominal wall defects.

### Population and clinical characteristics

We obtained the following demographic, obstetric, and neonatal characteristics: maternal age (categorised into: <20, 20–24, 25–29, 30–34, 35–39, and ≥40 years), ethnicity (categorised into: European, Mediterranean, Black, Asian, and other ethnicities)^32^, socio-economic status (SES; categorised into: high, middle, and low, and based on mean income level, the percentage of households with a low income, the percentage of inhabitants without a paid job, and the percentage of households with on average a low education in a postal code area)^33^, urbanisation level (rural or urban with a cut-off of 1,500 addresses per square kilometer at the postal code area level)^34^, parity (nulliparous vs. multiparous), pre-eclampsia (defined as diastolic blood pressure >90 mm Hg and proteinuria), mode of delivery (vaginal delivery vs. caesarean section), gestational age at delivery, sex of the newborn, birth weight, and whether the newborn was SGA or very SGA (below 10^th^ or 2.3^rd^ centile for gestational age according to parity, sex, and ethnic background with use of the Dutch PRN reference curves, respectively)^35^. Classification of ethnicity was based on previous research using PRN data which demonstrated important perinatal outcome differences across these groups^32^.

### Statistical analyses

Monthly incidences for each outcome were calculated to facilitate visualisation of temporal fluctuations and trends in changes of incidence levels. Interrupted time series logistic regression analyses with adjustment for potential confounders were performed to assess the associations between the 2004 and 2008 implementation of tobacco control policies and the odds of developing each of the primary and secondary outcome measures. The models accounted for the underlying temporal trend in odds of developing each outcome, and allowed for a sudden change (‘step change’) following the introduction of the policies over and above the underlying trend. Using Akaike’s and Schwarz’s Bayesian information criteria (AIC and BIC, respectively) we selected the optimal model among three options that varied in their modeling of the underlying temporal trend, which was modelled via linear, quadratic, and cubic B-splines, respectively, to capture possible non-linearity in the underlying time trends. A continuous time variable based on month of birth was used. By using a dummy variable for each combination of tobacco control policies (coded ‘0’ before and ‘1’ after introduction of the policies), the step changes for the odds of developing each outcome were modeled. A backward selection procedure was not applied and both intervention dummy variables were thus retained in each model. Babies were assigned to the period before or after the ban based on birth month. To account for seasonality, a categorical variable for month of birth was added.

The following categorical variables were included in the model to adjust for potential confounding at the individual level: maternal age, parity, urbanisation level, ethnicity, SES, pre-eclampsia, mode of delivery, and sex of the newborn. For the outcomes of stillbirth and perinatal mortality, mode of delivery was not included in the model. The primary analyses were performed using complete cases only (i.e. cases with complete data on all covariates in the analysis; 96% of cases. The numbers of cases with missing information for the different variables are shown in Supplementary Tables S1–S6.

For ease of textual presentation we report odds ratios (ORs) and their respective 95% confidence intervals (CIs) for the post-policy period versus the pre-policy period as % change in the post- as compared to the pre-policy period, using the following conversion:


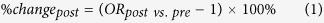


### Sensitivity analyses

For the primary outcome measures, we performed three sets of pre-specified sensitivity analyses to test the robustness of our results.

In the first set, we only included cases for whom pregnancy dating in the registry was recorded as ‘certain’ (93% of records). For the primary outcomes preterm birth and SGA, the model was thus re-run following the exclusion of cases in which gestational age was coded ‘not certain’. As this item had only been recorded from 2002 onwards, these analyses were restricted to the time period 2002–2011.

In the second set, cases with preterm birth before 26^+0^ weeks of gestation were excluded. Perinatal management in the Netherlands changed in 2010 with an increased active management for newborns born at the edge of viability (i.e. 24–25 weeks of gestation)^36^, which is likely to have affected perinatal mortality indicators and overall preterm birth rates. Although this effect was expected to be small, given the small proportion of preterm newborns born at this gestational age, the model was re-run for the outcome measures preterm birth and perinatal mortality.

In the third set, we stratified preterm births into spontaneous preterm births versus medically indicated (i.e. induced) preterm births to account for a potential change in clinical practice over time. Furthermore, using this analysis we could assess whether there was a differential association between the introduction of tobacco control policies and the type of preterm birth.

To test whether the findings were sensitive to missing data on individual-level covariates, multiple imputations using chained equations were performed, creating five unique datasets. We imputed the following variables: maternal age, ethnicity, SES, urbanisation level, parity, pre-eclampsia, mode of delivery, and sex of the newborn. Imputation was informed by both the outcome and the following predictor variables: gestational age and birth weight^37^.

We *a priori* planned to perform subgroup analyses according to maternal smoking status during pregnancy. However, maternal smoking during pregnancy was only recorded in <1% of the study population, which, based on prior studies, was considered to be a highly implausible smoking rate, indicating likely under-recording^38^.

### Counterfactual scenarios

To estimate the impact of the tobacco control policies in terms of the number of outcome events averted, counterfactual scenarios were developed for those outcomes for which a statistically significant association was observed. In these counterfactual scenarios, the odds of developing each outcome were estimated for the hypothetical situation of no policies having been introduced. The betas of each variable in the primary logistic regression models were used to calculate predicted values of the number of events, while setting the dummy variable of the policy of interest to ‘0’ for the entire study period. The estimated number of cases averted was then calculated by subtracting the actual number of events from the predicted number of events calculated based on the counterfactual model for each time interval.

All analyses were performed on a password-secured stand-alone computer located at PRN using Stata SE 12.0 (Statacorp, TX) and SAS version 9.3 (SAS Institute, Cary, NC). The Stata analysis code is provided as an online supplement.

### Ethical approval

The executive board of the PRN registry reviewed the protocol and gave their approval for the use of PRN data for this study (approval number 12.25). PRN data are anonymised; therefore according to Dutch law, formal ethical assessment of the study protocol was not needed.

## Results

**Figure 2:**
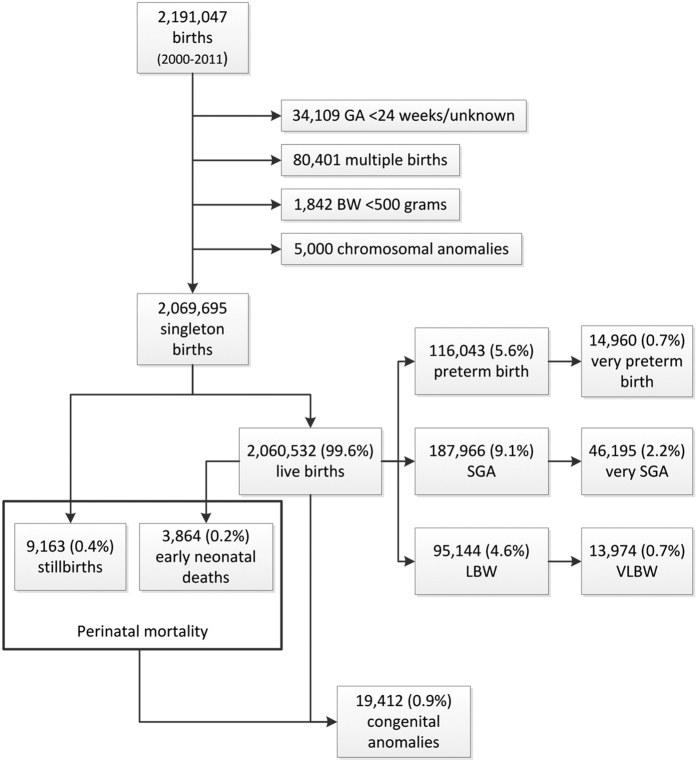
Flow chart of study population and primary and secondary outcomes. GA = gestational age; BW = birth weight; SGA = small for gestational age; LBW = low birth weight; VLBW = very low birth weight.

Between January 1^st^, 2000 and December 31^st^, 2011, 2,191,047 births were registered. We excluded newborns born at <24 weeks of gestation or with unknown gestational age (n = 34,109; 1.6%), multiple pregnancies (n = 80,401; 3.7%), newborns with a birth weight <500 grams (n = 1,842; 0.1%) and newborns with chromosomal anomalies (n = 5,000; 0.2%; Fig. 2). The resulting study population consisted of 2,069,695 newborns, among whom 9,163 (0.4%) stillbirths and 3,864 (0.2%) early neonatal deaths were identified. Among the 2,060,532 live-births, 116,043 (5.6%) preterm births and 187,966 (9.1%) SGA births were observed (Fig. 2). Furthermore, the study population included 14,960 (0.7%) cases of very preterm birth, 95,144 (4.6%) low birth weight newborns, including 13,974 (0.7%) with very low birth weight; 46,195 (2.2%) very SGA newborns, and 19,412 (0.9%) cases with congenital anomalies.

Demographic characteristics according to main and secondary outcomes, including the numbers of cases with missing data, are presented in Supplementary Tables S1–S6. All main outcomes were associated with low (<20 years) and high (≥40 years) maternal age, non-European ethnicities, low SES, and presence of pre-eclampsia. Living in an urban area was associated perinatal mortality as well as with being born SGA. Nulliparity was associated with perinatal mortality and preterm birth, but not with SGA birth. Perinatal mortality was associated with shorter duration of gestation and lower birth weight (Tables S1–3). Complete data on all covariates was available for 95–96% of the population, depending on the outcome of interest (Table 1). Visual assessment of actual and model-predicted monthly rates confirmed adequate modelling of temporal patterns in our primary outcomes (Supplemental Figure S2).

### Tobacco control policies and perinatal outcomes

The 2004 smoking ban in workplaces, mass media campaign and tobacco tax increase were not associated with statistically significant changes in the odds of developing any of the primary or secondary outcomes (Table 1). The July 2008 smoking ban in the hospitality industry, introduced in conjunction with another tax increase and mass media campaign, was associated with a statistically significant decrease in odds of SGA birth (−4.4%; 95% CI −6.4; −2.4, p < 0.001). The 2008 policy change was also associated with a statistically significant decreased odds of very preterm birth (−11.0%; 95% CI −18.6; −2.7, p = 0.01) and of very SGA birth (−7.7%; 95% CI −11.5; −3.8, p < 0.001).

Pre-specified sensitivity analyses confirmed the robustness of our findings, which were independent of health workers’ confidence in the gestational age estimate and of national consensus to lower the gestational age limit for active resuscitation (Table 2). Results were furthermore similar for indicated and spontaneous preterm births (Table 2), and did not materially change after multiple imputation to account for missing data (Table 1).

**Figure 3:**
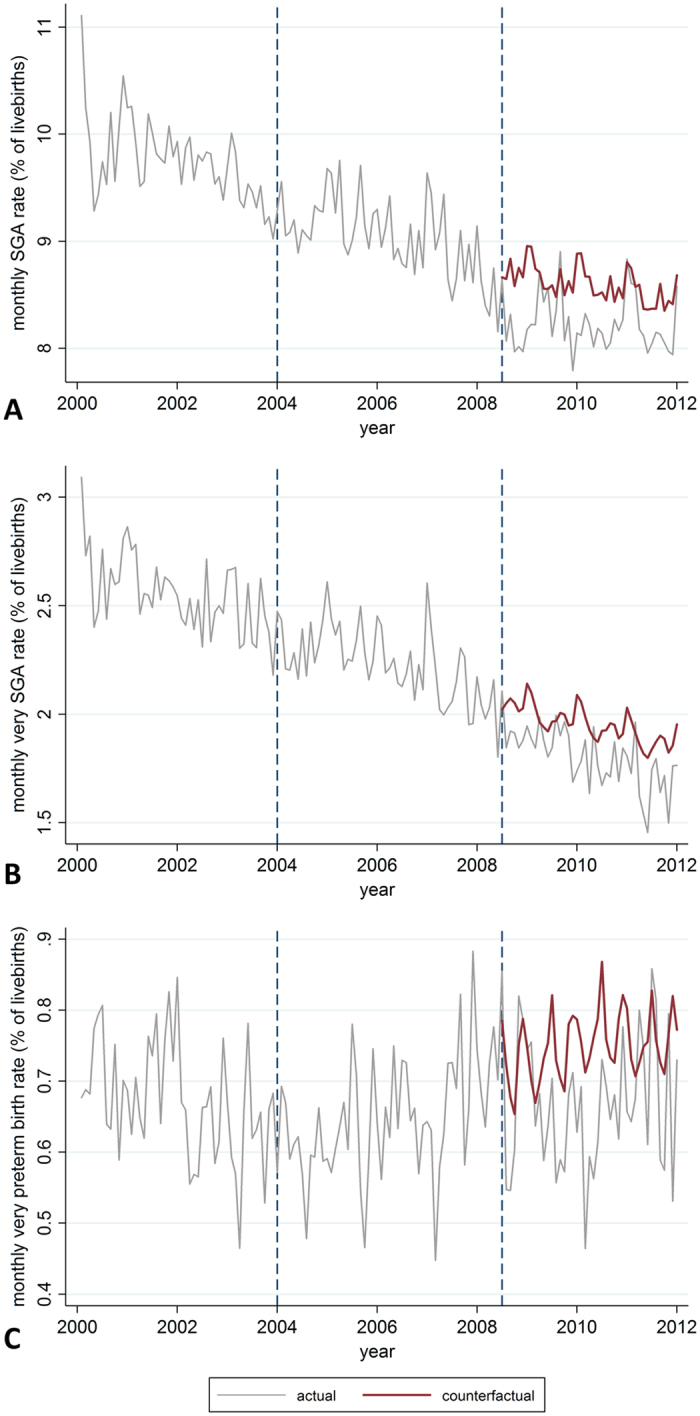
Actual and counterfactual monthly rates. Counterfactual rates are model predicted rates without tobacco control policy effects. Only outcomes for which significant changes were observed following tobacco control policies are shown. (**A**) small for gestational age (SGA); (**B**) very SGA; (**C**) very preterm birth. Dotted blue lines indicate timing of joint implementation of tobacco control policies. Note different scales on Y-axis.

Using a counterfactual scenario it was estimated that over the 3.5 years following the introduction of the 2008 tobacco control policies, 1,975 cases of SGA, including 847 very SGA births, and 459 cases of very preterm birth had been averted (Fig. 3).

## Discussion

In this quasi-experimental study we did not observe changes in key adverse pregnancy outcomes following introduction of a national smoke-free workplace law, mass media campaign and tobacco tax increase. Extending the legislation to include the hospitality industry, in conjunction with another tax increase and mass media campaign, was however associated with a significant reduction in the odds of being born SGA. We found no impact on overall preterm births or perinatal mortality.

With a study population of over two million births, this is one of the largest studies on this subject. We used a national dataset covering approximately 96% of all pregnancies in the Netherlands over a 12-year study period^19^. Our study period gives a balanced display of the period prior to, in between, and following the phased introduction of the tobacco control policies. Our findings were robust in several sensitivity analyses, including multiple imputation of missing covariates (4% of the study population).

Our study also has limitations. As with many governmental public health interventions, a randomised controlled trial was not feasible. A quasi-experimental approach is in such situations considered the optimal approach to evaluating their impact^39^. Inherent restrictions in definitely attributing causality apply however, and a control group in the usual sense is lacking^39^,^40^. We accounted for potential confounders; residual confounding by unmeasured variables, including other (public) health interventions or changes in perinatal management could have influenced our results. We are unaware of such sudden changes having co-occurred with timing of the policies, whereas any more gradual changes in odds of developing our study’s outcomes of interest over time are likely to have been captured by the accounting in our models for non-linear underlying temporal trends. The relatively larger tax increase accompanying the 2004 smoke-free law as compared to the 2008 ban suggests that the improvement in perinatal outcomes following the latter was mostly attributable to extension of the smoke-free law and the accompanying mass media campaign. However, because different tobacco control policies were introduced at the same time, we cannot differentiate between their individual effects. Another limitation of our study is the fact that in the PRN dataset some items – including maternal smoking status – are optional, resulting in many health care workers leaving those blank. Other covariates which may be potential confounders, such as maternal education and marital status, were not part of the dataset at all. We could not therefore take into account these variables in our analyses.

Our findings are consistent with previous studies demonstrating a decrease in SGA rates following introduction tobacco taxes and smoke-free legislation^7^,^13^,^14^,^17^, the latter being most pronounced among the subgroup of very SGA births^13^,^16^. Earlier studies of smoke-free legislation and tobacco tax increases indicate that reductions in both maternal smoking and maternal SHS exposure during pregnancy likely contributed to the associated perinatal health benefits, including reductions in SGA (Fig. 1)^7^,^8^,^13^,^14^,^17^,^41^,^42^. In the Netherlands a reduction in maternal smoking rates during pregnancy indeed coincided with the 2008 tobacco control policies^38^, however important under-recording of maternal smoking rates limited our ability to explore the potential underlying pathways in our study. At the same time it should be noted that this does not affect the validity of our conclusions regarding the association between the tobacco control policies and perinatal outcomes.

We did not observe a reduction in overall preterm births as seen in several studies following introduction of tobacco control policies^12^,^13^,^14^,^15^,^16^, however very preterm births (i.e. before 32 weeks of gestation) showed a significant drop. This is consistent with observational and intervention studies demonstrating a stronger effect of antenatal smoke exposure on very preterm as compared to overall preterm births^13^,^43^,^44^,^45^,^46^. Although the underlying mechanisms remain unclear, observational studies have demonstrated that antenatal smoke exposure has a stronger relationship with SGA birth than with overall preterm birth^42^,^47^,^48^, supporting the preferential impact of the policies on SGA observed in our study.

Comprehensive smoke-free laws are associated with larger health benefits than non-comprehensive laws in meta-regression analyses of adult studies^18^. Notably, larger health improvements are generally seen when workplace bans are scaled up to include restaurants and bars, as opposed to when workplace bans are implemented alone^18^. This is in agreement with our study, where perinatal health benefits were seen after the hospitality industry ban, but not after the introduction of the workplace ban, which may be due to a cumulative effect of both laws. The lack of impact of the workplace ban may relate to a considerable proportion of workplaces already being smoke-free before its implementation^24^. Extension of the law to bars and restaurants and the accompanying mass media campaign may have had are larger influence on awareness of the detrimental effects of antenatal smoke exposure and on social norms regarding maternal smoking and SHS exposure (Figure 1). Indeed, whereas maternal smoking rates during pregnancy in the Netherlands remained unchanged between 2003 and 2005, there was a 13% relative drop between 2007 and 2010^38^. Also, there was a 28% relative increase (p < 0.001) in the number of smokers having complete smoke-free homes between spring 2008 and spring 2009, thereby protecting non-smoking family members – including pregnant women – from SHS^49^.

Although a clinically relevant reduction in SGA births was observed following the 2008 smoking ban and tax increase, overall the benefits with regard to perinatal health were less pronounced than those observed in countries with more comprehensive smoke-free laws and better enforcement and compliance^11^,^12^,^13^,^17^. The smoke-free workplace law was faced with high levels of non-compliance as compared to international standards^30^,^50^. Also, a year after the introduction of the smoke-free hospitality law, 36% of smokers still noticed smoking in bars and cafes outside designated areas, which contrasts corresponding figures in other countries such as France (4.1%), the Republic of Ireland (5.3%) and the United Kingdom (UK) (1.7%)^27^,^51^. Smoking rates are higher and declines have been slower in the Netherlands than in the UK and Ireland^52^,^53^,^54^, where important perinatal health benefits of smoking bans have been described^11^,^13^,^17^. As the health impact of smoke-free legislation is thought to rely in part on norm spreading towards areas not typically covered, such as the home environment and private vehicles^55^,^56^, the relatively low proportion of Dutch smokers as opposed to smokers from other countries who consider SHS to be harmful to others and who worry about these effects, is of particular concern^57^,^58^. These figures suggest that at least part of the relative lack of impact of the Dutch smoke-free laws may be due to its lack of enforcement and compliance, although we cannot draw firm conclusions due to additional differences in study design, data collection methods, and population characteristics as compared to other studies.

In summary, implementation of a smoke-free law for the hospitality industry in the Netherlands, in conjunction with a tobacco tax increase and mass media campaign, was associated with an improvement of some perinatal outcomes, mainly a reduction in SGA births. At the same time, smoke-free laws in other countries with more comprehensive coverage, stronger enforcement, and better compliance have generally been associated with larger effect sizes and a wider array of perinatal health benefits^11^,^12^,^13^,^15^,^17^. Additional studies are needed to investigate whether the link between comprehensiveness of smoke-free laws and their health impact as observed in adults is also reproducible for babies and children^18^. This will provide highly relevant information to policy-makers worldwide who are planning implementation or extension of tobacco control policies to better protect the health of their population.

## Additional Information

**How to cite this article**: Peelen, M. J. *et al.* Tobacco control policies and perinatal health: a national quasi-experimental study. *Sci. Rep.* **6**, 23907; doi: 10.1038/srep23907 (2016).

## Supplementary Material

Supplementary Information

## Figures and Tables

**Table 1:** Associations between tobacco control policies and primary and secondary outcomes.

Primary analyses								Multiple imputation						
Primary outcomes	N	Smoke-free workplace + tax increase + mass media campaign (2004)			Smoke-free bars and restaurants + tax increase + mass media campaign (2008)			N	Smoke-free workplace + tax increase + mass media campaign (2004)			Smoke-free bars and restaurants + tax increase + mass media campaign (2008)		
		OR	95% CI	p-value	OR	95% CI	p-value		OR	95% CI	p-value	OR	95% CI	p-value
Perinatal mortality	1,980,727	0.98	0.91–1.06	0.67	0.94	0.87–1.02	0.15	2,069,695	0.99	0.92–1.06	0.67	0.93	0.86–1.01	0.15
Preterm birth	1,972,163	1.01	0.98–1.04	0.61	0.99	0.96–1.03	0.71	2,060,532	1.01	0.98–1.05	0.61	1.00	0.97–1.03	0.71
SGA	1,972,157	0.99	0.97–1.01	0.18	0.96	0.94–0.98	<0.001	2,059,805	0.99	0.97–1.01	0.18	0.96	0.94–0.98	<0.001
Secondary outcomes														
Stillbirth	1,983,761	0.99	0.91–1.08	0.84	0.97	0.88–1.06	0.51							
Early neonatal mortality	1,972,163	0.97	0.84–1.12	0.69	0.88	0.76–1.02	0.08							
Very preterm birth	1,972,163	0.94	0.86–1.03	0.18	0.89	0.81–0.97	0.01							
LBW	1,972,163	1.00	0.97–1.04	0.88	0.97	0.94–1.01	0.15							
VLBW	1,972,163	0.94	0.86–1.03	0.19	0.94	0.85–1.03	0.20							
Very SGA	1,972,157	1.02	0.98–1.06	0.29	0.92	0.89–0.96	<0.001							
Congenital anomalies	1,983,761	1.01	0.94–1.08	0.84	0.98	0.91–1.06	0.61							

Odds ratios represent the odds for each outcome in the period after the introduction of each set of tobacco control policies versus the preceding period. Primary analyses were adjusted for non-linear time trends, month, maternal age, ethnicity, socioeconomic status, level of urbanisation, parity, preeclampsia, fetal sex, and caesarean section. For the primary analyses only cases with no missing variables were included. OR = odds ratio; CI = confidence interval; SGA = small for gestational age; LBW = low birth weight; VLBW = very low birth weight.

**Table 2:** Association between tobacco control policies and primary outcomes: sensitivity analyses.

	Smoke-free workplace + tax increase + mass media campaign (2004)			Smoke-free bars and restaurants + tax increase + mass media campaign (2008)		
	OR	95% CI	p-value	OR	95% CI	p-value
Set 1						
Preterm birth	1.00	0.96–1.05	0.92	1.00	0.96–1.03	0.90
SGA	0.98	0.96–1.00	0.11	0.95	0.93–0.98	<0.001
Set 2						
Preterm birth	1.01	0.98–1.04	0.53	0.99	0.96–1.03	0.76
Perinatal mortality	0.99	0.91–1.07	0.81	0.94	0.87–1.03	0.18
Set 3						
Spontaneous preterm birth	1.01	0.98–1.05	0.58	0.99	0.95–1.02	0.50
Medically indicated preterm birth	0.99	0.92–1.06	0.67	1.02	0.96–1.09	0.56

Set 1 included only cases with a ‘certain’ gestational age; analyses restricted to 2002–2011 time period. Set 2 included only cases ≥26 + 0 weeks of gestation. Set 3 included a subdivision into spontaneous and medically indicated preterm birth. OR = odds ratio; CI = confidence interval; SGA = small for gestational age.
