# Supplementary material for: Tobacco control policies and perinatal health: a national quasi-experimental study
Source: Sci Rep. 2016 Apr 22;6:23907. doi: 10.1038/srep23907 (PMC4840332; doi:10.1038/srep23907)
Supplement: Supplementary Information [file srep23907-s1.pdf]

Supplementary Information

Tobacco control policies and perinatal health: a national quasi-experimental study

Myrthe J PEELEN; Aziz SHEIKH; Marjolein KOK; Petra HAJENIUS; Luc J ZIMMERMANN; Boris W KRAMER; Chantal W HUKKELHOVEN; Irwin K REISS; Ben W MOL; Jasper V BEEN

Supplementary Figures: page 2-4

Supplementary Tables: page 5-16

Supplementary Stata Code: page 17-64

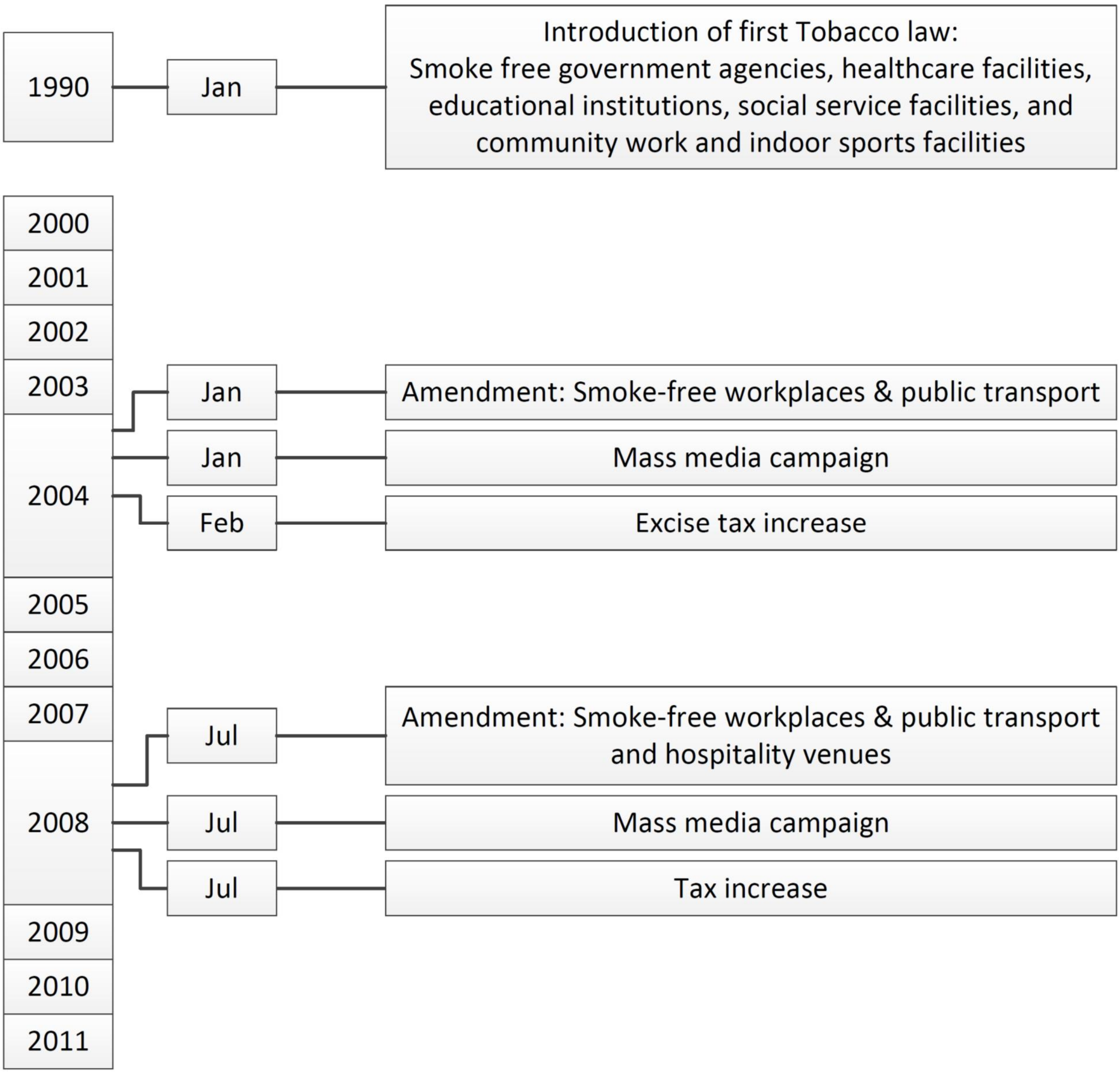

Figure S1. Timeline of key tobacco control policies evaluated.

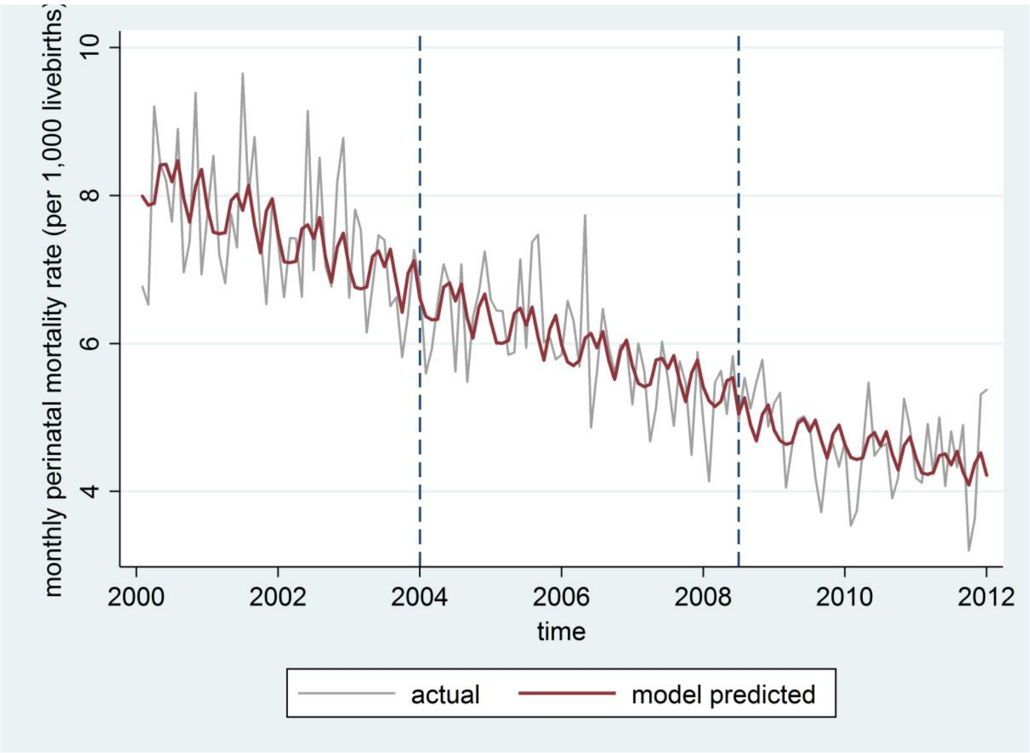
monthly perinatal mortality rate (per 1,000 livebirths)
10
8
6
4
2000
2002
2004
2006
2008
2010
2012
time
actual
model predicted

A

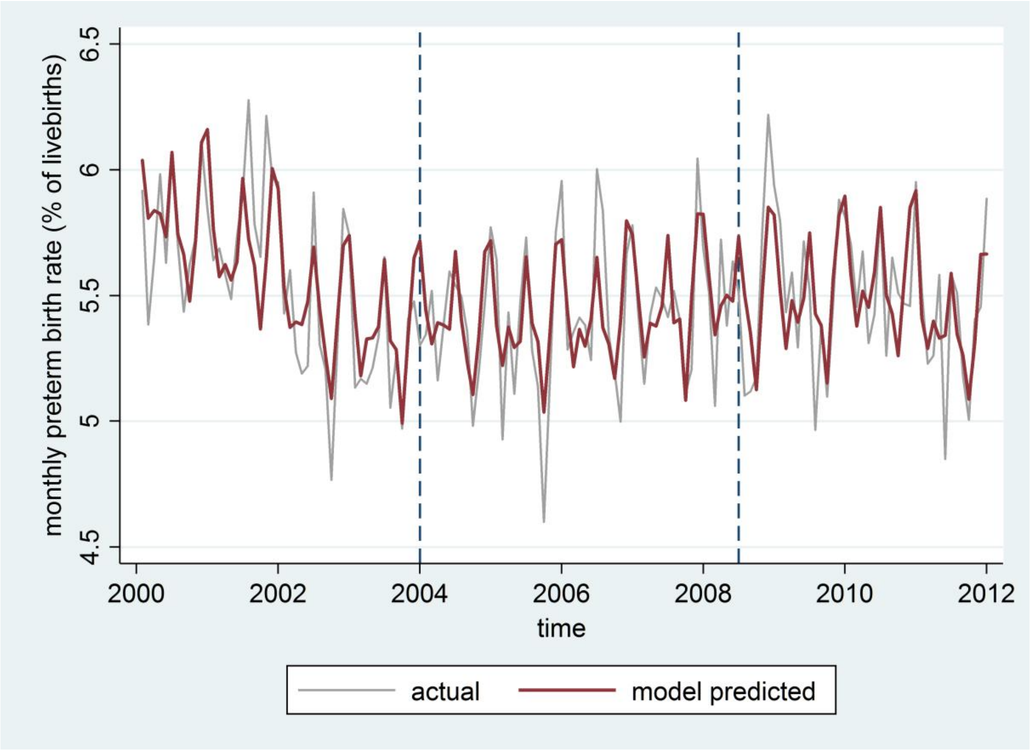
monthly preterm birth rate (% of livebirths)
6.5
6
5.5
5
4.5
2000
2002
2004
2006
2008
2010
2012
time
actual
model predicted

B

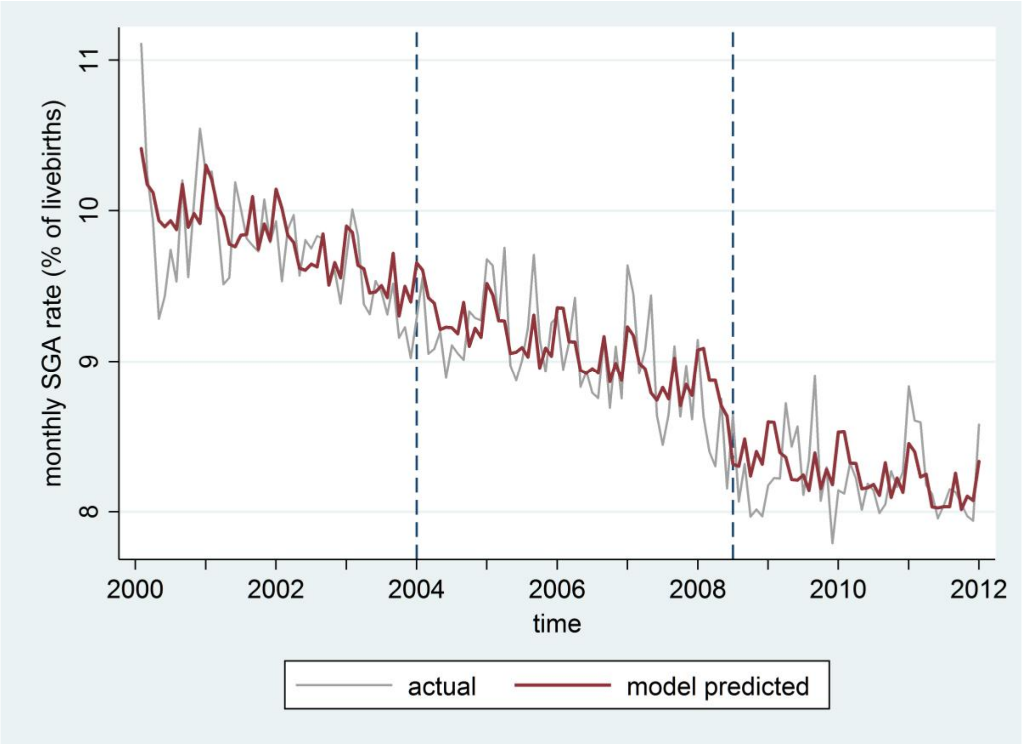

C

Figure S2. Actual and model predicted monthly rates for the primary outcomes. A: perinatal mortality; B: preterm birth; C: small for gestational age (SGA). Dotted blue lines indicate timing of joint implementation of tobacco control policies. Note different scales on Y-axis.

Table S1 Demographic characteristics according to different mortality categories

	Live-births (n=2,060,532)	Perinatal mortality		
		All perinatal deaths (n= 13,027)	Stillbirths (n=9,163)	Early neonatal deaths (n= 3,864)
Maternal age (years)				
<20	33,225 (1.6)	342 (2.6)	235 (2.6)	107 (2.8)
20-24	211,108 (10.3)	1,563 (12.0)	1,106 (12.1)	457 (11.8)
25-29	601,947 (29.2)	3,511 (27.0)	2,427 (26.5)	1,084 (28.1)
30-34	796,252 (38.6)	4,524 (34.7)	3,151 (34.4)	1,373 (35.5)
35-39	362,337 (17.6)	2,511 (19.3)	1,818 (19.8)	693 (17.9)
≥40	55,408 (2.7)	575 (4.4)	425 (4.6)	150 (3.9)
Missing	255 (0.0)	1 (0.0)	1 (0.0)	0 (0)
Ethnicity				
European	1,717,431 (83.3)	10,066 (77.6)	7,034 (77.1)	3,032 (78.8)
Mediterranean	158,073 (7.7)	1,305 (10.1)	923 (10.1)	382 (9.9)
Black	49,762 (2.4)	594 (4.6)	431 (4.7)	163 (4.2)
Asian	62,531 (3.0)	456 (3.5)	344 (3.8)	112 (2.9)
Other	61,488 (3.0)	551 (4.2)	391 (4.3)	160 (4.2)
Missing	11,247 (0.6)	55 (0.4)	40 (0.4)	15 (0.4)
Socioeconomic status				
Low	627,660 (30.5)	4,537 (35.4)	3,255 (36.1)	1,282 (33.9)
Intermediate	924,397 (44.9)	5,671 (44.3)	3,980 (44.1)	1,691 (44.7)
High	471,872 (22.9)	2,604 (20.3)	1,793 (19.9)	811 (21.4)
Missing	36,603 (1.8)	215 (1.7)	135 (1.5)	80 (2.1)
Urbanization level				
Rural	1,229,181 (59.7)	7,473 (57.7)	5,246 (57.5)	2,227 (58.2)
Urban	825,592 (40.1)	5,476 (42.3)	3,873 (42.5)	1,603 (41.9)
Missing	5,759 (0.3)	78 (0.6)	44 (0.5)	34 (0.9)
Parity				
Nulliparous	950,574 (46.1)	6,619 (50.8)	4,713 (51.4)	1,906 (49.3)
Multiparous	1,109,822 (53.9)	6,407 (49.2)	4,449 (48.6)	1,958 (50.7)
Missing	136 (0.0)	1 (0.0)	1 (0.0)	0 (0)
Pre-eclampsia				
No	1,982,004 (98.0)	11,845 (95.3)	8,328 (95.1)	3,517 (95.8)
Yes	40,436 (2.0)	581 (4.7)	427 (4.9)	154 (4.2)
Missing	38,092 (1.8)	601 (4.6)	408 (4.5)	193 (5.0)
Mode of delivery				
Vaginal delivery	1,757,133 (85.5)	11,247 (86.6)	8,676 (95.0)	2,571 (66.9)
Cesarean section	298,949 (14.5)	1,733 (13.4)	460 (5.0)	1,273 (33.1)
Missing	4,450 (0.2)	47 (0.4)	27 (0.3)	20 (0.5)

Gestational age at delivery				
<32 weeks	14,960 (0.7)	4,524 (34.7)	3,058 (33.4)	1,466 (37.9)
32-36 weeks	101,083 (4.9)	3,018 (23.2)	2,265 (24.7)	753 (19.5)
≥37 weeks	1,944,489 (94.4)	5,485 (42.1)	3,840 (41.9)	1,645 (42.6)
Sex				
Male	1,057,315 (51.3)	6,953 (53.5)	4,812 (52.6)	2,141 (55.7)
Female	1,002,633 (48.7)	6,039 (46.5)	4,333 (47.4)	1,706 (44.4)
Missing	584 (0.0)	35 (0.3)	18 (0.2)	17 (0.4)
Birth weight (grams)				
<1,500	13,974 (0.7)	4,681 (35.9)	3,247 (35.4)	1,434 (37.1)
1,500-2,499	81,170 (3.9)	2,985 (22.9)	2,213 (24.2)	772 (20.0)
≥2,500	1,965,388 (95.4)	5,361 (41.2)	3,703 (40.4)	1,658 (42.9)
Missing	0 (0.0)	0 (0.0)	0 (0.0)	0 (0.0)
SGA (<p10)	187,966 (9.1)	3,458 (26.5)	2,670 (29.2)	788 (20.5)
Very SGA (<p2.3)	46,195 (2.2)	1,525 (11.7)	1,152 (12.6)	373 (9.7)
Missing birth weight centile	727 (0.0)	36 (0.3)	19 (0.2)	17 (0.4)
Congenital anomalies	19,005 (0.9)	1,062 (8.2)	407 (4.4)	655 (17.0)

SGA: small for gestational age

Percentages in parentheses relate to all newborns with non-missing data for that variable. Percentages for missing data relate to the full sample.

Table S2 Demographic characteristics according to preterm birth categories

	Term birth (≥37 weeks) (n=1,944,489)	Preterm birth (<37 weeks) (n=116,043)	Very preterm birth (<32 weeks) (n=14,960)
Maternal age (years)			
<20	30,454 (1.6)	2,771 (2.4)	500 (3.3)
20-24	197,566 (10.2)	13,542 (11.7)	2,032 (13.6)
25-29	566,446 (29.1)	35,501 (30.6)	4,409 (29.5)
30-34	754,641 (38.8)	41,611 (35.9)	5,061 (33.8)
35-39	343,365 (17.7)	18,972 (16.4)	2,431 (16.3)
≥40	51,775 (2.7)	3,633 (3.1)	527 (3.5)
Missing	242 (0.0)	13 (0.0)	0 (0.0)
Ethnicity			
European	1,621,583 (83.9)	95,848 (82.9)	11,723 (78.9)
Mediterranean	150,383 (7.8)	7,690 (6.7)	1,153 (7.8)
Black	45,780 (2.4)	3,982 (3.4)	775 (5.2)
Asian	58,248 (3.0)	4,283 (3.7)	488 (3.3)
Other	57,657 (3.0)	3,831 (3.3)	722 (4.8)
Missing	10,838 (0.6)	409 (0.4)	99 (0.7)
Socioeconomic status			
Low	589,595 (30.9)	38,065 (33.5)	5,407 (37.3)
Intermediate	873,383 (45.7)	51,014 (44.9)	6,227 (43.0)
High	447,230 (23.4)	24,642 (21.7)	2,850 (19.7)
Missing	34,281 (1.8)	2,322 (2.0)	476 (3.2)
Urbanization level			
Rural	1,161,218 (59.9)	67,963 (58.9)	8,170 (55.5)
Urban	778,140 (40.1)	47,452 (41.1)	6,541 (44.5)
Missing	5,131 (0.3)	628 (0.5)	249 (1.7)
Parity			
Nulliparous	881,361 (45.3)	69,213 (59.7)	9,202 (61.5)
Multiparous	1,062,997 (54.7)	46,825 (40.4)	5,757 (38.5)
Missing	131 (0.0)	5 (0.0)	1 (0.0)
Pre-eclampsia			
No	1,883,060 (98.5)	98,944 (89.0)	10,875 (80.2)
Yes	28,142 (1.5)	12,294 (11.1)	2,690 (19.8)
Missing	33,287 (1.7)	4,805 (4.1)	1,395 (9.3)
Onset of delivery			
Spontaneous	1,535,177 (79.2)	84,097 (72.8)	8,619 (58.3)
Medically indicated	403,512 (20.8)	31,461 (27.2)	6,170 (41.7)
Missing	5,800 (0.3)	485 (0.4)	171 (1.1)
Mode of delivery			

Vaginal delivery	1,674,901 (86.3)	82,232 (71.0)	7,485 (50.2)
Cesarean section	265,387 (13.7)	33,562 (29.0)	7,418 (49.8)
Missing	4,201 (0.2)	249 (0.2)	57 (0.4)
Sex			
Male	993,180 (51.1)	64,135 (55.3)	8,370 (56.0)
Female	950,785 (48.9	51,848 (44.7)	6,566 (44.0)
Missing	524 (0.0)	60 (0.1)	24 (0.2)
Birth weight (grams)			
<1,500	165 (0.0)	13,809 (11.9)	10,845 (72.5)
1,500-2,499	34,154 (1.8)	47,016 (40.5)	4,115 (27.5)
≥2,500	1,910,170 (98.2)	55,218 (47.6)	0 (0.0)
Missing	0 (0.0)	0 (0.0)	0 (0.0)
SGA (<p10)	177,270 (9.1)	10,696 (9.2)	1,207 (8.1)
Very SGA (<p2.3)	43,238 (2.2)	2,957 (2.6)	186 (1.3)
Missing birth weight centile	662 (0.0)	65 (0.1)	25 (0.2)
Congenital anomalies	15,774 (0.8)	3,231 (2.8)	860 (5.8)

SGA: small for gestational age

Percentages in parentheses relate to all newborns with non-missing data for that variable. Percentages for missing data relate to the full sample. Only live-births were included.

Table S3 Demographic characteristics according to small-for-gestational-age categories

	No SGA (n= 1,871,839)	SGA (n= 187,966)	Very SGA (n= 46,195)	Missing birth weight centile (n= 727)
Maternal age (years)				
<20	28,834 (1.5)	4,378 (2.3)	1,103 (2.4)	13 (1.8)
20-24	187,205 (10.0)	23,813 (12.7)	5,948 (12.9)	90 (12.6)
25-29	547,729 (29.3)	54,002 (28.7)	13,006 (28.2)	216 (30.3)
30-34	729,596 (39.0)	66,389 (35.3)	15,940 (34.5)	267 (37.4)
35-39	328,896 (17.6)	33,331 (17.7)	8,487 (18.4)	110 (15.4)
≥40	49,357 (2.6)	6,033 (3.2)	1,705 (3.7)	18 (2.5)
Missing	222 (0.0)	20 (0.0)	6 (0.0)	13 (1.8)
Ethnicity				
European	1,569,451 (84.3)	147,428 (78.9)	36,383 (79.2)	552 (79.0)
Mediterranean	141,176 (7.6)	16,823 (9.0)	3,957 (8.6)	74 (10.6)
Black	41,606 (2.2)	8,133 (4.4)	2,094 (4.6)	23 (3.3)
Asian	55,693 (3.0)	6,816 (3.7)	1,602 (3.5)	22 (3.1)
Other	53,829 (2.9)	7,631 (4.1)	1,917 (4.2)	28 (4.0)
Missing	10,084 (0.5)	1,135 (0.6)	242 (0.5)	28 (3.9)
Socioeconomic status				
Low	559,430 (30.4)	67,945 (36.7)	17,485 (38.4)	285 (41.2)
Intermediate	844,239 (45.9)	79,873 (43.2)	19,477 (42.8)	285 (41.2)
High	434,571 (23.6)	37,180 (20.1)	8,545 (18.8)	121 (17.5)
Missing	33,599 (1.8)	2,968 (1.6)	688 (1.5)	36 (5.0)
Urbanization level				
Rural	1,123,932 (60.2)	104,877 (55.9)	25,713 (55.8)	372 (52.6)
Urban	742,663 (39.8)	82,594 (44.1)	20,358 (44.2)	335 (47.4)
Missing	5,244 (0.3)	495 (0.3)	124 (0.3)	20 (2.8)
Parity				
Nulliparous	863,016 (46.1)	87,304 (46.5)	21,897 (47.4)	254 (43.0)
Multiparous	1,008,823 (53.9)	100,662 (53.6)	24,298 (52.6)	337 (57.0)
Missing	0 (0.0)	0 (0.0)	0 (0.0)	136 (18.7)
Pre-eclampsia				
No	1,805,366 (98.2)	175,934 (95.9)	42,707 (95.1)	704 (98.5)
Yes	32,878 (1.8)	7,547 (4.1)	2,182 (4.9)	11 (1.5)
Missing	33,595 (1.8)	4,485 (2.4)	1,306 (2.8)	12 (1.7)
Mode of delivery				
Vaginal delivery	1,601,138 (85.7)	155,321 (82.8)	35,328 (76.6)	674 (92.7)
Cesarean section	266,575 (14.3)	32,321 (17.2)	10,790 (23.4)	53 (7.3)
Missing	4,126 (0.2)	324 (0.2)	77 (0.2)	0 (0.0)
Gestational age at delivery				

<32 weeks	13,728 (0.7)	1,207 (0.6)	186 (0.4)	25 (3.4)
32-36 weeks	91,554 (4.9)	9,489 (5.1)	2,771 (6.0)	40 (5.5)
≥37 weeks	1,766,557 (94.4)	177,270 (94.3)	43,238 (93.6)	662 (91.1)
Missing	0 (0.0)	0 (0.0)	0 (0.0)	0 (0.0)
Sex				
Male	961,284 (51.4)	95,967 (51.1)	23,664 (51.2)	64 (44.8)
Female	910,555 (48.6)	91,999 (48.9)	22,531 (48.8)	79 (55.2)
Missing	0 (0.0)	0 (0.0)	0 (0.0)	584 (80.3)
Birth weight (grams)				
<1,500	10,468 (0.6)	3,481 (1.9)	1,337 (2.9)	25 (3.4)
1,500-2,499	42,775 (2.3)	38,349 (20.4)	20,876 (45.2)	46 (6.3)
≥2,500	1,818,596 (97.2)	146,136 (77.8)	23,982 (51.9)	656 (90.2)
Missing	0 (0.0)	0 (0.0)	0 (0.0)	0 (0.0)
Congenital anomalies	15,994 (0.9)	2,991 (1.6)	1,138 (2.5)	20 (2.8)

SGA: small for gestational age

Percentages in parentheses relate to all newborns with non-missing data for that variable. Percentages for missing data relate to the full sample. Only live births were included.

Table S4 Demographic characteristics according to birth weight categories

	Birth weight ≥ 2500 g (n= 1,965,388)	Low birth weight (n= 95,144)	Very low birth weight (n= 13,974)
Maternal age (years)			
<20	30,441 (1.6)	2,784 (2.9)	464 (3.3)
20-24	198,406 (10.1)	12,702 (13.4)	1,990 (14.2)
25-29	573,478 (29.2)	28,469 (29.9)	4,071 (29.1)
30-34	763,925 (38.9)	32,327 (34.0)	4,591 (32.9)
35-39	346,679 (17.6)	15,658 (16.5)	2,341 (16.8)
≥40	52,212 (2.7)	3,196 (3.4)	516 (3.7)
Missing	247 (0.0)	8 (0.0)	1 (0.0)
Ethnicity			
European	1,641,446 (84.0)	75,985 (80.2)	10,805 (77.9)
Mediterranean	151,561 (7.8)	6,512 (6.9)	1,100 (7.9)
Black	45,776 (2.3)	3,986 (4.2)	790 (5.7)
Asian	57,821 (3.0)	4,710 (5.0)	505 (3.6)
Other	57,940 (3.0)	3,548 (3.7)	673 (4.9)
Missing	10,844 (0.6)	403 (0.4)	101 (0.7)
Socioeconomic status			
Low	593,398 (30.7)	34,262 (36.7)	5,228 (38.6)
Intermediate	883,878 (45.8)	40,519 (43.5)	5,759 (42.5)
High	453,393 (23.5)	18,479 (19.8)	2,569 (19.0)
Missing	34,719 (1.8)	1,884 (2.0)	418 (3.0)
Urbanization level			
Rural	1,175,824 (60.0)	53,357 (56.4)	7,534 (54.8)
Urban	784,333 (40.0)	41,259 (43.6)	6,223 (45.2)
Missing	5,231 (0.3)	528 (0.6)	217 (1.6)
Parity			
Nulliparous	891,706 (45.4)	58,868 (61.9)	8,834 (63.2)
Multiparous	1,073,550 (54.6)	36,272 (38.1)	5,140 (36.8)
Missing	132 (0.0)	4 (0.0)	0 (0.0)
Pre-eclampsia			
No	1,904,081 (98.6)	77,923 (86.0)	9,118 (72.4)
Yes	27,719 (1.4)	12,717 (14.0)	3,469 (27.6)
Missing	33,588 (1.7)	4,504 (4.7)	1,387 (9.9)
Mode of delivery			
Vaginal delivery	1,695,230 (86.4)	61,903 (65.2)	5,209 (37.4)
Cesarean section	265,913 (13.6)	33,036 (34.8)	8,714 (62.6)
Missing	4,245 (0.2)	205 (0.2)	51 (0.4)
Gestational age at delivery			

<32 weeks	0 (0.0)	14,960 (15.7)	10,845 (77.6)
32-36 weeks	55,218 (2.8)	45,865 (48.2)	2,964 (21.2)
≥37 weeks	1,910,170 (97.2)	34,319 (36.1)	165 (1.2)
Missing	0 (0.0)	0 (0.0)	0 (0.0)
Sex			
Male	1,011,583 (51.5)	45,732 (48.1)	7,155 (51.3)
Female	953,288 (48.5)	49,345 (51.9)	6,794 (48.7)
Missing	517 (0.0)	67 (0.1)	25 (0.2)
SGA (<p10)	146,136 (7.4)	41,830 (44.0)	3,481 (25.0)
Very SGA (<p2.3)	23,982 (1.2)	22,213 (23.4)	1,337 (9.6)
Missing birth weight centile	656 (0.0)	71 (0.1)	25 (0.2)
Congenital anomalies	15,886 (0.8)	3,119 (3.3)	867 (6.2)

SGA: small for gestational age

Percentages in parentheses relate to all newborns with non-missing data for that variable. Percentages for missing data relate to the full sample. Only live births were included.

Table S5 Demographic characteristics for congenital anomalies

	No congenital anomalies (n=2,050,283)	Congenital anomalies (n=19,412)
Maternal age (years)		
<20	33,026 (1.6)	434 (2.2)
20-24	209,990 (10.2)	2,224 (11.5)
25-29	598,706 (29.2)	5,668 (29.2)
30-34	792,386 (38.7)	7,017 (36.2)
35-39	360,753 (17.6)	3,402 (17.5)
≥40	55,168 (2.7)	665 (3.4)
Missing	254 (0.0)	2 (0.0)
Ethnicity		
European	1,708,810 (83.8)	15,655 (81.0)
Mediterranean	157,366 (7.7)	1,630 (8.4)
Black	49,450 (2.4)	743 (3.8)
Asian	62,239 (3.1)	636 (3.3)
Other	61,209 (3.0)	670 (3.5)
Missing	11,209 (0.6)	78 (0.4)
Socioeconomic status		
Low	624,472 (31.0)	6,443 (33.8)
Intermediate	919,655 (45.7)	8,722 (45.8)
High	469,778 (23.3)	3,887 (20.4)
Missing	36,378 (1.8)	360 (1.9)
Urbanization level		
Rural	1,222,948 (59.8)	11,479 (59.4)
Urban	821,618 (40.2)	7,847 (40.6)
Missing	5,717 (0.3)	86 (0.4)
Parity		
Nulliparous	945,551 (46.1)	9,736 (50.2)
Multiparous	1,104,596 (53.9)	9,675 (49.8)
Missing	136 (0.0)	1 (0.0)
Pre-eclampsia		
No	1,972,163 (98.0)	18,169 (96.5)
Yes	40,211 (2.0)	652 (3.5)
Missing	37,909 (1.9)	591 (3.0)
Mode of delivery		
Vaginal delivery	1,750,802 (85.6)	15,007 (77.5)
Cesarean section	295,193 (14.4)	4,346 (22.5)
Missing	4,418 (0.2)	59 (0.3)
Gestational age at delivery		

<32 weeks	16,981 (0.8)	1,037 (5.3)
32-36 weeks	100,840 (4.9)	2,508 (12.9)
≥37 weeks	1,932,462 (94.3)	15,867 (81.7)
Missing	0 (0.0)	0 (0.0)
Sex		
Male	1,051,102 (51.3)	11,025 (56.9)
Female	998,600 (48.7)	8,366 (43.1)
Missing	581 (0.0)	21 (0.1)
Birth weight (grams)		
<1,500	16,158 (0.8)	1,063 (5.5)
1,500-2,499	80,998 (4.0)	2,385 (12.3)
≥2,500	1,953,127 (95.3)	15,964 (82.2)
Missing	0 (0.0)	0 (0.0)
SGA (<p10)	187,500 (9.2)	3,136 (16.2)
Very SGA (<p2.3)	46,125 (2.3)	1,222 (6.3)
Missing birth weight centile	724 (0.0)	22 (0.1)

SGA: small for gestational age

Percentages in parentheses relate to all newborns with non-missing data for that variable. Percentages for missing data relate to the full sample. Both live-births and stillbirths were included.

Table S6 Demographic characteristics of preterm births by onset of labor

	Spontaneous preterm birth (n= 84,097)	Medically indicated preterm birth (n= 31,461)	Unknown (n= 485)
Maternal age (years)			
<20	2,200 (2.6)	557 (1.8)	14 (2.9)
20-24	10,028 (11.9)	3,448 (11.0)	66 (13.6)
25-29	26,406 (31.4)	8,953 (28.5)	142 (29.3)
30-34	30,384 (36.1)	11,067 (35.2)	160 (33.0)
35-39	12,882 (15.3)	6,004 (19.1)	86 (17.7)
≥40	2,185 (2.6)	1,431 (4.6)	17 (3.5)
Missing	12 (0.0)	1 (0.0)	0 (0.0)
Ethnicity			
European	69,838 (83.3)	25,662 (81.9)	348 (72.3)
Mediterranean	5,586 (6.7)	2,065 (6.6)	39 (8.1)
Black	2,531 (3.0)	1,408 (4.5)	43 (8.9)
Asian	3,225 (3.9)	1,042 (3.3)	16 (3.3)
Other	2,632 (3.1)	1,164 (3.7)	35 (7.3)
Missing	285 (0.3)	120 (0.4)	4 (0.8)
Socioeconomic status			
Low	27,252 (33.1)	10,602 (34.4)	211 (44.9)
Intermediate	37,084 (45.0)	13,755 (44.7)	175 (37.2)
High	18,124 (22.0)	6,434 (20.9)	84 (17.9)
Missing	1,637 (2.0)	670 (2.1)	15 (3.1)
Urbanization level			
Rural	49,486 (59.1)	18,266 (58.5)	211 (43.9)
Urban	34,217 (40.9)	12,965 (41.5)	270 (56.1)
Missing	394 (0.5)	230 (0.7)	4 (0.8)
Parity			
Nulliparous	51,112 (60.8)	17,846 (56.7)	255 (52.6)
Multiparous	32,980 (39.2)	13,615 (43.3)	230 (47.4)
Missing	5 (0.0)	0 (0.0)	0 (0.0)
Pre-eclampsia			
No	80,403 (98.3)	18,217 (62.6)	324 (89.5)
Yes	1,362 (1.7)	10,894 (37.4)	38 (10.5)
Missing	2,332 (2.8)	2,350 (7.5)	123 (25.4)
Mode of delivery			
Vaginal delivery	74,250 (88.5)	7,659 (24.4)	323 (71.8)
Cesarean section	9650 (11.5)	23,785 (75.6)	127 (28.2)
Missing	197 (0.2)	17 (0.1)	35 (7.2)

Gestational age at delivery			
<32 weeks	8,619 (10.2)	6,170 (19.6)	171 (35.3)
32-36 weeks	75,478 (89.8)	25,291 (80.4)	314 (64.7)
Missing	0 (0.0)	0 (0.0)	0 (0.0)
Sex			
Male	47,757 (56.8)	16,124 (51.3)	254 (52.5)
Female	36,298 (43.2)	15,320 (48.7)	230 (47.5)
Missing	42 (0.1)	17 (0.1)	1 (0.2)
Birth weight (grams)			
<1,500	5,856 (7.0)	7,821 (24.9)	132 (27.2)
1,500-2,499	32,238 (38.3)	14,552 (46.3)	226 (46.6)
≥2,500	46,003 (54.7)	9,088 (28.9)	127 (26.2)
SGA (<p10)	3,341 (4.0)	7,299 (23.2)	56 (11.6)
Very SGA (<p2.3)	676 (0.8)	2,262 (7.2)	19 (3.9)
Missing birth weight centile	47 (0.1)	17 (0.1)	1 (0.2)
Congenital anomalies	2,092 (2.5)	1,108 (3.5)	31 (6.4)

SGA: small for gestational age

Percentages in parentheses relate to all newborns with non-missing data for that variable. Percentages for missing data relate to the full sample. Only live births were included.

```
********************************************************
* FORMAT MASTERFILE FROM YEAR EXTRACTS OF RELEVANT DATA *
********************************************************

*note1: variables necessary for analyses were extracted in SAS by perinatal registry
*       team and stored in yearly extracts

*note2: all data-checks (coding etc) and data cleaning were performed by perinatal
*       registry team in SAS

clear
set memory 1g

log using "formatting masterfile.smcl", replace

*import 2000 data from SAS into Stata and save as masterfile
usesas using "jaar2000.sas7bdat", clear
save "masterfile.dta", replace

*import 2001-2011 data from SAS into Stata and append to masterfile
*2001
usesas using "jaar2001.sas7bdat", clear
append using "masterfile.dta", force
save "masterfile.dta", replace

*2002
usesas using "jaar2002.sas7bdat", clear
append using "masterfile.dta", force
save "masterfile.dta", replace

*2003
usesas using "jaar2003.sas7bdat", clear
append using "masterfile.dta", force
save "masterfile.dta", replace

*2004
usesas using "jaar2004.sas7bdat", clear
append using "masterfile.dta", force
save "masterfile.dta", replace

*2005
usesas using "jaar2005.sas7bdat", clear
append using "masterfile.dta", force
save "masterfile.dta", replace


*2006
usesas using "jaar2006.sas7bdat", clear
```

```
append using "masterfile.dta", force
save "masterfile.dta", replace

*2007
usesas using "jaar2007.sas7bdat", clear
append using "masterfile.dta", force
save "masterfile.dta", replace

*2008
usesas using "jaar2008.sas7bdat", clear
append using "masterfile.dta", force
save "masterfile.dta", replace

*2009
usesas using "jaar2009.sas7bdat", clear
append using "masterfile.dta", force
save "masterfile.dta", replace

*2010
usesas using "jaar2010.sas7bdat", clear
append using "masterfile.dta", force
save "masterfile.dta", replace

*2011
usesas using "jaar2011.sas7bdat", clear
append using "masterfile.dta", force
save "masterfile.dta", replace

*generate unique identifier
sort jaar weekgeboorte
*note 'jaar' = year; 'weekgeboorte' = week of birth
generate id=[_n]


*generate variable names and labels for variables

*gestational age certain yes/no
generate termdatcert = v_zek

*preterm birth yes/no
rename vg_zwdwk ga
label variable ga "gestational age"
generate preterm = 0
replace preterm = 1 if ga<37
replace preterm = . if ga == .
label define yn 0 "no" 1 "yes"
label values preterm yn
```

```
*birth weight continuous
rename vgn_gew bw
label variable bw "birth weight"

*mode of delivery
rename vg_wijzebar mod
label variable mod "mode of delivery"
label define mod 1 "spontaneous delivery" 2 "instrumental delivery" 3 "elective CS" 4 "emergency CS"
label values mod mod

*parity (nulli/multiparous)
generate parity = 0
replace parity = 1 if vg_pari >1
replace parity = . if vg_pari == .
replace parity = . if vg_pari <0
replace parity = . if vg_pari >25
label define parity 0 "nulliparous" 1 "multiparous"
label values parity parity

*year (continuous)
rename jaar year

*maternal age (categorised)
rename vg_lftv ma
label variable ma "maternal age"
generate macat = 1
replace macat = 2 if ma > 19
replace macat = 3 if ma > 24
replace macat = 4 if ma > 29
replace macat = 5 if ma > 34
replace macat = 6 if ma > 39
replace macat = . if ma == .
replace macat = . if ma <10
replace macat = . if ma >60
label variable macat "maternal age category"
label define macat 1 "<20y" 2 "20-24y" 3 "25-29y" 4 "30-34y" 5 "35-39y" 6 ">=40y"
label values macat macat

*gestational age (categorised)
rename zwduurcat gacat
label variable gacat "gestational age category"
label define gacat 1 "<28w" 2 "28-31w" 3 "32-36w" 4 ">=37w"
label values gacat gacat

*socioeconomic status (categorised)
label variable ses "socioeconomic status"
label define ses 1 "high" 2 "intermediate" 3 "low"
label values ses ses
```

```
*ethnicity (categorised)
rename etniciteit ethn
label variable ethn "ethnicity"
label define ethn 1 "European" 2 "Mediterranean" 3 "Black" 4 "Asian" 5 "Other"
label values ethn ethn

*sex (male/female/unclear)
rename vgn_gesl sex
label variable sex "sex"
label define sex 1 "male" 2 "female" 3 "unclear"
label values sex sex

*mortality (categorised by timing)
rename vgn_mortk5 mortality
label variable mortality "mortality"
replace mortality = 1 if mortality == 2
replace mortality = . if mortality == 9
replace mortality = . if mortality == 99
label define mortality 0 "stillbirth" 1 "early neonatal death" 3 "late neonatal death" 4 "alive at 28 days"
label values mortality mortality

*birth weight (categorised)
rename vgn_gewcat bwcat
label variable bwcat "birth weight category"
label define bwcat 1 "<1500g" 2 "1500-2499g" 3 "2500-3999g" 4 ">=4000g"
label values bwcat bwcat

*chromosomal anomalies (yes/no)
rename chromosomaleafw chroman
label variable chroman "chromosomal anomalies"
label values chroman yn

*congenital anomalies (yes/no)
rename congafw congan
label variable congan "congenital anomalies"
label values congan yn

*preeclampsia (yes/no)
label variable pe "preeclampsia"
label values pe yn

*small for gestational age (yes/no)
rename p10 sga
label variable sga "SGA"
label values sga yn

*very small for gestational age (yes/no)
```

```
rename p233 vsga
label variable vsga "very SGA"
label values vsga yn

*week of birth (continuous)
rename weekgeboorte wob
label variable wob "week of birth"

*month of birth (categorised)
rename maandgeboorte mob
label variable mob "month of birth"
label define month 1 "January" 2 "February" 3 "March" 4 "April" 5 "May" 6 "June" 7 "July" 8 "August" ///
	9 "September" 10 "October" 11 "November" 12 "December"
label values mob month

*urbanisation level (dichotomised)
generate urban = .
replace urban = 1 if urbanisatie == 1
replace urban = 1 if urbanisatie == 2
replace urban = 0 if urbanisatie == 3
replace urban = 0 if urbanisatie == 4
replace urban = 0 if urbanisatie == 5
label variable urban "urbanisation"
label define urban 0 "rural" 1 "urban"
label values urban urban

*maternal smoking during pregnancy (yes/no)
rename roken smok
label variable smok "smoking during pregnancy"
label values smok yn

*drop redundant variables
drop vg_pari park2 vg_etn vgn_geencgm urbanisatie

save "masterfile.dta", replace

log close


***********************************************
* ANALYSES FOR BASELINE CHARACTERISTICS TABLES *
***********************************************

log using "demographic counts.smcl", replace
use "masterfile.dta", clear

*prepare counts for chromosomal anomalies
```

```
	*generate count variable for base population
	generate count = 1
	egen totpop = sum(count)

	*generate count variable for study population (excluding chromosomal anomalies)
	generate studycount = 1
	replace studycount = 0 if chroman == 1

	*calculate population size + numbers and % chromosomal anomalies
	egen totstudypop = sum(studycount)
	egen totchroman = sum(chroman)
	generate totnochroman = totpop-totchroman
	generate percchroman = totchroman/totpop*100
	generate percnochroman = totnochroman/totpop*100

	*report numbers and % chromosomal anomalies
	title "chromosomal anomalies"
	summarize totpop totstudypop totchroman percchroman percnochroman

*prepare counts for congenital anomalies

	*exclude chromosomal anomalies
	replace congan = . if chroman == 1

	*calculate population size + numbers and % congenital anomalies
	egen totcongan = sum(congan)
	generate totnocongan = totstudypop-totcongan
	generate perccongan = totcongan/totstudypop*100
	generate percnocongan = totnocongan/totstudypop*100

	*report numbers and % congenital anomalies
	title "congenital anomalies"
	summarize totcongan totnocongan perccongan percnocongan
```

```
*prepare counts for mortality outcomes

	*stillbirth

	*generate count variable for stillbirths
	generate sb = 0
	replace sb = 1 if mortality == 0

	*exclude chromosomal anomalies
	replace sb = . if chroman == 1

	*calculate population size + numbers and % stillbirths
	egen totsb = sum(sb)
	generate totnosb = totstudypop-totsb
	generate percsb = totsb/totstudypop*100
	generate percnosb = totnosb/totstudypop*100

	*report numbers and % stillbirths
	title "stillbirth"
	summarize totsb totnosb percsb percnosb

	*neonatal mortality

	*generate count variable for neonatal mortality
	generate neonmort = 0
	replace neonmort = 1 if mortality == 1
	replace neonmort = 1 if mortality == 3

	*exclude chromosomal anomalies
	replace neonmort = . if chroman == 1

	*calculate population size + numbers and % neonatal mortality
	egen totneonmort = sum(neonmort)
	generate totnoneonmort = totstudypop-totneonmort
	generate percneonmort = totneonmort/totstudypop*100
	generate percnoneonmort = totnoneonmort/totstudypop*100

	*report numbers and % neonatal mortality
	title "neonatal mortality"
	summarize totneonmort totnoneonmort percneonmort percnoneonmort

	*early neonatal mortality

	*generate count variable for early neonatal mortality
	generate eneonmort = 0
	replace eneonmort = 1 if mortality == 1

	*exclude chromosomal anomalies
```

```
replace eneonmort = . if chroman == 1

*calculate population size + numbers and % early neonatal mortality
egen toteneonmort = sum(eneonmort)
generate totnoeneonmort = totstudypop-toteneonmort
generate perceneonmort = toteneonmort/totstudypop*100
generate percnoeneonmort = totnoeneonmort/totstudypop*100

*report numbers and % early neonatal mortality
title "early neonatal mortality"
summarize toteneonmort totnoeneonmort perceneonmort percnoeneonmort

*late neonatal mortality

*generate count variable for late neonatal mortality
generate lneonmort = 0
replace lneonmort = 1 if mortality == 3

*exclude chromosomal anomalies
replace lneonmort = . if chroman == 1

*calculate population size + numbers and % late neonatal mortality
egen totlneonmort = sum(lneonmort)
generate totnolneonmort = totstudypop-totlneonmort
generate perclneonmort = totlneonmort/totstudypop*100
generate percnolneonmort = totnolneonmort/totstudypop*100

*report numbers and % late neonatal mortality
title "late neonatal mortality"
summarize totlneonmort totnolneonmort perclneonmort percnolneonmort
```

```
*prepare counts for birth weight outcomes among livebirths

    *exclude chromosomal anomalies and stillbirths
    drop if chroman == 1
    drop if sb == 1

    *calculate and report number and % of missing birth weight
    generate bwc = 1
    replace bwc = 0 if bwcat == .
    egen totbwc = sum(bwc)
    egen totlb = sum(count)
    generate totbwm = totlb-totbwc
    generate percbwc = totbwc/totlb*100
    generate percbwm = totbwm/totlb*100
    title "missing birth weight"
    summarize totlb totbwc totbwm percbwc percbwm
```

```
        *exclude cases with missing birth weight
        drop if bwc == 0

        *calculate and report number and % of very low birth weight
        generate vlbw = 0
        replace vlbw = 1 if bwcat == 1
        egen totvlbw = sum(vlbw)
        generate percvlbw = totvlbw/totbwc*100
        title "very low birth weight"
        summarize totvlbw percvlbw

        *calculate and report number and % of low birth weight
        generate lbw = 0
        replace lbw = 1 if bwcat == 1
        replace lbw = 1 if bwcat == 2
        egen totlbw = sum(lbw)
        generate perclbw = totlbw/totbwc*100
        title "low birth weight"
        summarize totlbw perclbw

*prepare counts for gestational age categories among livebirths
use "masterfile.dta", clear

        *exclude stillbirths and chromosomal anomalies
        generate sb = 0
        replace sb = 1 if mortality == 0
        replace sb = . if chroman == 1
        drop if chroman == 1
        drop if sb == 1

        *define base population
        generate count = 1
        egen totpop = sum(count)

        *calculate and report number and % of missing gestational age
        generate gac = 1
        replace gac = 0 if ga == .
        egen totgac = sum(gac)
        generate totgam = totpop-totgac
        generate percgac = totgac/totpop*100
        generate percgam = totgam/totpop*100
        title "missing gestational age"
        summarize totpop totgac totgam percgac percgam

        *exclude cases with missing gestational age
        drop if gac == 0

        *calculate and report number and % of very preterm birth
```

```
generate vptb = 0
replace vptb = 1 if ga < 32
egen totvptb = sum(vptb)
generate percvpt = totvptb/totpop*100
title "very preterm birth"
summarize totvptb percvpt

*calculate and report number and % of very preterm birth
generate ptb = 0
replace ptb = 1 if ga < 37
egen totptb = sum(ptb)
generate percpt = totptb/totpop*100
title "preterm birth"
summarize totptb percpt
```

```
*prepare counts for birth weight centile categories among livebirths
use "masterfile.dta", clear

    *exclude stillbirths and chromosomal anomalies
    generate sb = 0
    replace sb = 1 if mortality == 0
    replace sb = . if chroman == 1
    drop if chroman == 1
    drop if sb == 1

    *define base population
    generate count = 1
    egen totpop = sum(count)

    *calculate and report number and % of missing birth weight centile
    generate sgac = 1
    replace sgac = 0 if sga == .
    egen totsgac = sum(sgac)
    generate totsgam = totpop-totsgac
    generate percsgac = totsgac/totpop*100
    generate percsgam = totsgam/totpop*100
    title "missing birth weight centile"
    summarize totpop totsgac totsgam percsgac percsgam


    *exclude cases with missing birth weight centile
    drop if sgac == 0

    *calculate and report number and % of SGA births
    egen totsga = sum(sga)
    generate percsga = totsga/totsgac*100
    title "SGA"
    summarize totsgac totsga percsga
```

```
	*calculate and report number and % of very SGA births
	egen totvsga = sum(vsga)
	generate percvsga = totvsga/totsgac*100
	title "very SGA"
	summarize totsgac totvsga percvsga

*prepare demographics for still vs livebirths
use "masterfile.dta", clear

	*generate stillbirth indicator
	generate sb = 0
	replace sb = 1 if mortality == 0
	replace sb = . if chroman == 1

	*exclude chromosomal anomalies
	drop if chroman == 1

	*tabulate demographoic variables according to stillbirth yes/no,
	*		reporting numbers and % with and without cases with missing data
tab2 sb ethn, row missing
tab2 sb ethn, row
tab2 sb ses, row missing
tab2 sb ses, row
tab2 sb urban, row missing
tab2 sb urban, row
tab2 sb macat, row missing
tab2 sb macat, row
tab2 sb smok, row missing
tab2 sb smok, row
tab2 sb parity, row missing
tab2 sb parity, row
tab2 sb mod, row missing
tab2 sb mod, row
tab2 sb pe, row missing
tab2 sb pe, row
tab2 sb gacat, row missing
tab2 sb gacat, row
tab2 sb bwcat, row missing
tab2 sb bwcat, row
tab2 sb sga, row missing
tab2 sb sga, row
tab2 sb vsga, row missing
tab2 sb vsga, row
tab2 sb sex, row missing
tab2 sb sex, row
tab2 sb congan, row missing
tab2 sb congan, row
```

```
*prepare demographics for congenital anomalies
use "masterfile.dta", clear

	*exclude chromosomal anomalies
	drop if chroman == 1

	*tabulate demographoic variables according to congenital anomalies yes/no,
	*	reporting numbers and % with and without cases with missing data
tab2 congan ethn, row missing
tab2 congan ethn, row
tab2 congan ses, row missing
tab2 congan ses, row
tab2 congan urban, row missing
tab2 congan urban, row
tab2 congan macat, row missing
tab2 congan macat, row
tab2 congan smok, row missing
tab2 congan smok, row
tab2 congan parity, row missing
tab2 congan parity, row
tab2 congan mod, row missing
tab2 congan mod, row
tab2 congan pe, row missing
tab2 congan pe, row
tab2 congan gacat, row missing
tab2 congan gacat, row
tab2 congan bwcat, row missing
tab2 congan bwcat, row
tab2 congan sga, row missing
tab2 congan sga, row
tab2 congan vsga, row missing
tab2 congan vsga, row
tab2 congan sex, row missing
tab2 congan sex, row

*prepare demographics for neonatal mortality
use "masterfile.dta", clear

	*exclude stillbirths and congenital anomalies
	generate sb = 0
	replace sb = 1 if mortality == 0
	replace sb = . if chroman == 1
	drop if chroman == 1
	drop if sb == 1

	*generate neonatal mortality indicator
	generate neonmort = 0
```

```
	replace neonmort = 1 if mortality == 1
	replace neonmort = 1 if mortality == 3

	*tabulate demographoic variables according to neonatal mortality yes/no,
	*	reporting numbers and % with and without cases with missing data
tab2 neonmort ethn, row missing
tab2 neonmort ethn, row
tab2 neonmort ses, row missing
tab2 neonmort ses, row
tab2 neonmort urban, row missing
tab2 neonmort urban, row
tab2 neonmort macat, row missing
tab2 neonmort macat, row
tab2 neonmort smok, row missing
tab2 neonmort smok, row
tab2 neonmort parity, row missing
tab2 neonmort parity, row
tab2 neonmort mod, row missing
tab2 neonmort mod, row
tab2 neonmort pe, row missing
tab2 neonmort pe, row
tab2 neonmort gacat, row missing
tab2 neonmort gacat, row
tab2 neonmort bwcat, row missing
tab2 neonmort bwcat, row
tab2 neonmort sga, row missing
tab2 neonmort sga, row
tab2 neonmort vsga, row missing
tab2 neonmort vsga, row
tab2 neonmort sex, row missing
tab2 neonmort sex, row
tab2 neonmort congan, row missing
tab2 neonmort congan, row

*prepare demographics for neonatal mortality groups
use "masterfile.dta", clear

	*exclude stillbirths and chromosomal anomalies
	generate sb = 0
	replace sb = 1 if mortality == 0
	replace sb = . if chroman == 1
	drop if chroman == 1
	drop if sb == 1

	*tabulate demographoic variables according to early and late neonatal mortality yes/no,
	*	reporting numbers and % with and without cases with missing data
tab2 mortality ethn, row missing
tab2 mortality ethn, row
```

```
tab2 mortality ses, row missing
tab2 mortality ses, row
tab2 mortality urban, row missing
tab2 mortality urban, row
tab2 mortality macat, row missing
tab2 mortality macat, row
tab2 mortality smok, row missing
tab2 mortality smok, row
tab2 mortality parity, row missing
tab2 mortality parity, row
tab2 mortality mod, row missing
tab2 mortality mod, row
tab2 mortality pe, row missing
tab2 mortality pe, row
tab2 mortality gacat, row missing
tab2 mortality gacat, row
tab2 mortality bwcat, row missing
tab2 mortality bwcat, row
tab2 mortality sga, row missing
tab2 mortality sga, row
tab2 mortality vsga, row missing
tab2 mortality vsga, row
tab2 mortality sex, row missing
tab2 mortality sex, row
tab2 mortality congan, row missing
tab2 mortality congan, row

*produce demographics for low birth weight
use "masterfile.dta", clear

        *exclude stillbirths and chromosomal anomalies
        generate sb = 0
        replace sb = 1 if mortality == 0
        replace sb = . if chroman == 1
        drop if chroman == 1
        drop if sb == 1

        *identify low birth weight cases
        generate lbw = 0
        replace lbw = 1 if bwcat == 1
        replace lbw = 1 if bwcat == 2

        *tabulate demographoic variables according to low birth weigth yes/no,
        *       reporting numbers and % with and without cases with missing data
tab2 lbw ethn, row missing
tab2 lbw ethn, row
tab2 lbw ses, row missing
tab2 lbw ses, row
```

```
tab2 lbw urban, row missing
tab2 lbw urban, row
tab2 lbw macat, row missing
tab2 lbw macat, row
tab2 lbw smok, row missing
tab2 lbw smok, row
tab2 lbw parity, row missing
tab2 lbw parity, row
tab2 lbw mod, row missing
tab2 lbw mod, row
tab2 lbw pe, row missing
tab2 lbw pe, row
tab2 lbw gacat, row missing
tab2 lbw gacat, row
tab2 lbw bwcat, row missing
tab2 lbw bwcat, row
tab2 lbw sga, row missing
tab2 lbw sga, row
tab2 lbw vsga, row missing
tab2 lbw vsga, row
tab2 lbw sex, row missing
tab2 lbw sex, row
tab2 lbw congan, row missing
tab2 lbw congan, row

*produce demographics for very low birth weight
use "masterfile.dta", clear

        *exclude stillbirths and chromosomal anomalies
        generate sb = 0
        replace sb = 1 if mortality == 0
        replace sb = . if chroman == 1
        drop if chroman == 1
        drop if sb == 1

        *identify very low birth weight cases
        generate vlbw = 0
        replace vlbw = 1 if bwcat == 1

        *tabulate demographhoic variables according to very low birth weight yes/no,
        *       reporting numbers and % with and without cases with missing data
tab2 vlbw ethn, row missing
tab2 vlbw ethn, row
tab2 vlbw ses, row missing
tab2 vlbw ses, row
tab2 vlbw urban, row missing
tab2 vlbw urban, row
tab2 vlbw macat, row missing
```

```
tab2 vlbw macat, row
tab2 vlbw smok, row missing
tab2 vlbw smok, row
tab2 vlbw parity, row missing
tab2 vlbw parity, row
tab2 vlbw mod, row missing
tab2 vlbw mod, row
tab2 vlbw pe, row missing
tab2 vlbw pe, row
tab2 vlbw gacat, row missing
tab2 vlbw gacat, row
tab2 vlbw bwcat, row missing
tab2 vlbw bwcat, row
tab2 vlbw sga, row missing
tab2 vlbw sga, row
tab2 vlbw vsga, row missing
tab2 vlbw vsga, row
tab2 vlbw sex, row missing
tab2 vlbw sex, row
tab2 vlbw congan, row missing
tab2 vlbw congan, row

*produce demographics for preterm birth
use "masterfile.dta", clear

        *exclude stillbirths and chromosomal anomalies
        generate sb = 0
        replace sb = 1 if mortality == 0
        replace sb = . if chroman == 1
        drop if chroman == 1
        drop if sb == 1

        *tabulate demographoic variables according to preterm birth yes/no,
        *       reporting numbers and % with and without cases with missing data
tab2 preterm ethn, row missing
tab2 preterm ethn, row
tab2 preterm ses, row missing
tab2 preterm ses, row
tab2 preterm urban, row missing
tab2 preterm urban, row
tab2 preterm macat, row missing
tab2 preterm macat, row
tab2 preterm smok, row missing
tab2 preterm smok, row
tab2 preterm parity, row missing
tab2 preterm parity, row
tab2 preterm mod, row missing
tab2 preterm mod, row
```

```
tab2 preterm pe, row missing
tab2 preterm pe, row
tab2 preterm gacat, row missing
tab2 preterm gacat, row
tab2 preterm bwcat, row missing
tab2 preterm bwcat, row
tab2 preterm sga, row missing
tab2 preterm sga, row
tab2 preterm vsga, row missing
tab2 preterm vsga, row
tab2 preterm sex, row missing
tab2 preterm sex, row
tab2 preterm induced, row missing
tab2 preterm induced, row
tab2 preterm congan, row missing
tab2 preterm congan, row

*produce demographics for spontaneous vs induced preterm birth
use "masterfile.dta", clear

        *exclude stillbirths and chromosomal anomalies
        generate sb = 0
        replace sb = 1 if mortality == 0
        replace sb = . if chroman == 1
        drop if chroman == 1
        drop if sb == 1
        drop if preterm == 0

        *tabulate demographoic variables according to preterm birth yes/no,
        *       stratified by whether preterm birth was spontaneous or indicated,
        *       reporting numbers and % with and without cases with missing data
tab2 induced ethn, row missing
tab2 induced ethn, row
tab2 induced ses, row missing
tab2 induced ses, row
tab2 induced urban, row missing
tab2 induced urban, row
tab2 induced macat, row missing
tab2 induced macat, row
tab2 induced smok, row missing
tab2 induced smok, row
tab2 induced parity, row missing
tab2 induced parity, row
tab2 induced mod, row missing
tab2 induced mod, row
tab2 induced pe, row missing
tab2 induced pe, row
tab2 induced gacat, row missing
```

```
tab2 induced gacat, row
tab2 induced bwcat, row missing
tab2 induced bwcat, row
tab2 induced sga, row missing
tab2 induced sga, row
tab2 induced vsga, row missing
tab2 induced vsga, row
tab2 induced sex, row missing
tab2 induced sex, row
tab2 induced congan, row missing
tab2 induced congan, row

*produce demographics for spontaneous vs induced very preterm birth

        *tabulate demographoic variables according to very preterm birth yes/no,
        *       stratified by whether very preterm birth was spontaneous or indicated,
        *       reporting numbers and % with and without cases with missing data
generate vptb = 0
replace vptb = 1 if gacat == 1
replace vptb = 1 if gacat == 2
keep if vptb == 1
tab2 induced ethn, row missing
tab2 induced ethn, row
tab2 induced ses, row missing
tab2 induced ses, row
tab2 induced urban, row missing
tab2 induced urban, row
tab2 induced macat, row missing
tab2 induced macat, row
tab2 induced smok, row missing
tab2 induced smok, row
tab2 induced parity, row missing
tab2 induced parity, row
tab2 induced mod, row missing
tab2 induced mod, row
tab2 induced pe, row missing
tab2 induced pe, row
tab2 induced gacat, row missing
tab2 induced gacat, row
tab2 induced bwcat, row missing
tab2 induced bwcat, row
tab2 induced sga, row missing
tab2 induced sga, row
tab2 induced vsga, row missing
tab2 induced vsga, row
tab2 induced sex, row missing
tab2 induced sex, row
tab2 induced congan, row missing
```

```
tab2 induced congan, row

*produce demographics for very preterm birth
use "masterfile.dta", clear

        *exclude stillbirths and chromosomal anomalies
        generate sb = 0
        replace sb = 1 if mortality == 0
        replace sb = . if chroman == 1
        drop if chroman == 1
        drop if sb == 1

        *generate very preterm birth indicator
        generate vptb = 0
        replace vptb = 1 if gacat == 1
        replace vptb = 1 if gacat == 2

        *tabulate demographic variables according to very preterm birth yes/no,
        *       reporting numbers and % with and without cases with missing data
tab2 vptb ethn, row missing
tab2 vptb ethn, row
tab2 vptb ses, row missing
tab2 vptb ses, row
tab2 vptb urban, row missing
tab2 vptb urban, row
tab2 vptb macat, row missing
tab2 vptb macat, row
tab2 vptb smok, row missing
tab2 vptb smok, row
tab2 vptb parity, row missing
tab2 vptb parity, row
tab2 vptb mod, row missing
tab2 vptb mod, row
tab2 vptb pe, row missing
tab2 vptb pe, row
tab2 vptb gacat, row missing
tab2 vptb gacat, row
tab2 vptb bwcat, row missing
tab2 vptb bwcat, row
tab2 vptb sga, row missing
tab2 vptb sga, row
tab2 vptb vsga, row missing
tab2 vptb vsga, row
tab2 vptb sex, row missing
tab2 vptb sex, row
tab2 vptb induced, row missing
tab2 vptb induced, row
tab2 vptb congan, row missing
```

```
tab2 vptb congan, row

*produce demographics for missing birth weight centile
use "masterfile.dta", clear

        *exclude stillbirths and chromosomal anomalies
        generate sb = 0
        replace sb = 1 if mortality == 0
        replace sb = . if chroman == 1
        drop if chroman == 1
        drop if sb == 1

        *keep only cases with missing birth weight centile
        keep if sga == .

        *tabulate demographic variables for cases with missing birth weight centile,
        *       reporting numbers and % with and without cases with missing data
tab2 sga ethn, row missing
tab2 sga ethn, row
tab2 sga ses, row missing
tab2 sga ses, row
tab2 sga urban, row missing
tab2 sga urban, row
tab2 sga macat, row missing
tab2 sga macat, row
tab2 sga smok, row missing
tab2 sga smok, row
tab2 sga parity, row missing
tab2 sga parity, row
tab2 sga mod, row missing
tab2 sga mod, row
tab2 sga pe, row missing
tab2 sga pe, row
tab2 sga gacat, row missing
tab2 sga gacat, row
tab2 sga bwcat, row missing
tab2 sga bwcat, row
tab2 sga sex, row missing
tab2 sga sex, row
tab2 sga congan, row missing
tab2 sga congan, row

*produce demographics for SGA
use "masterfile.dta", clear

        *exclude stillbirths, chromosomal anomalies,
        *       and cases with missing birth weight centile
        generate sb = 0
```

```
	replace sb = 1 if mortality == 0
	replace sb = . if chroman == 1
	drop if chroman == 1
	drop if sb == 1
	drop if sga == .
	drop if vsga == .

	*tabulate demographic variables according to SGA yes/no,
	*	reporting numbers and % with and without cases with missing data
tab2 sga ethn, row missing
tab2 sga ethn, row
tab2 sga ses, row missing
tab2 sga ses, row
tab2 sga urban, row missing
tab2 sga urban, row
tab2 sga macat, row missing
tab2 sga macat, row
tab2 sga smok, row missing
tab2 sga smok, row
tab2 sga parity, row missing
tab2 sga parity, row
tab2 sga mod, row missing
tab2 sga mod, row
tab2 sga pe, row missing
tab2 sga pe, row
tab2 sga gacat, row missing
tab2 sga gacat, row
tab2 sga bwcat, row missing
tab2 sga bwcat, row
tab2 sga sga, row missing
tab2 sga sga, row
tab2 sga vsga, row missing
tab2 sga vsga, row
tab2 sga sex, row missing
tab2 sga sex, row
tab2 sga congan, row missing
tab2 sga congan, row

*produce demographics for vSGA
use "masterfile.dta", clear

	*exclude stillbirths, chromosomal anomalies,
	*	and cases with missing birth weight centile
	generate sb = 0
	replace sb = 1 if mortality == 0
	replace sb = . if chroman == 1
	drop if chroman == 1
	drop if sb == 1
```

```
    drop if sga == .
    drop if vsga == .

    *tabulate demographic variables according to very SGA yes/no,
    *       reporting numbers and % with and without cases with missing data
tab2 vsga ethn, row missing
tab2 vsga ethn, row
tab2 vsga ses, row missing
tab2 vsga ses, row
tab2 vsga urban, row missing
tab2 vsga urban, row
tab2 vsga macat, row missing
tab2 vsga macat, row
tab2 vsga smok, row missing
tab2 vsga smok, row
tab2 vsga parity, row missing
tab2 vsga parity, row
tab2 vsga mod, row missing
tab2 vsga mod, row
tab2 vsga pe, row missing
tab2 vsga pe, row
tab2 vsga gacat, row missing
tab2 vsga gacat, row
tab2 vsga bwcat, row missing
tab2 vsga bwcat, row
tab2 vsga sga, row missing
tab2 vsga sga, row
tab2 vsga vsga, row missing
tab2 vsga vsga, row
tab2 vsga sex, row missing
tab2 vsga sex, row
tab2 vsga congan, row missing
tab2 vsga congan, row

log close


**************************************
* MAIN ANALYSES OF PRIMARY OUTCOMES *
**************************************

set more off
log using "analyses-primary outcomes.smcl", replace
use "masterfile.dta", clear

*drop chromosomal anomalies
drop if chroman == 1
```

```
*generate primary outcome variables

*perinatal mortality
generate perimort = 0
replace perimort = 1 if mortality == 1
	*exclude cases of stillbirth or missing info on mortality status
	replace perimort = 1 if mortality == 0
	replace perimort = . if mortality == .

*preterm birth: exclude cases of stillbirth or missing info on mortality status
replace preterm = . if mortality == 0
replace preterm = . if mortality == .
```

```
*SGA: exclude cases of stillbirth or missing info on mortality status
replace sga = . if mortality == 0
replace sga = . if mortality == .

*re-categorise mode of delivery from categorical into dichotomous
*           (caesarean section yes/no)
generate cs = 0
replace cs = 1 if mod == 3
replace cs = 1 if mod == 4
replace cs = . if mod == .

*generate continuous time variable in months
generate time = (year-2000)*12 + mob

*generate ban dummy to model 2004 ban
generate ban1 = 0
replace ban1 = 1 if time>47
label variable ban1 "2004 ban"

*generate ban dummy to model 2008 ban
generate ban2 = 0
replace ban2 = 1 if time>101
label variable ban2 "2008 ban"

*generate bsplines to model (non-linear) time trends
*linear
bspline, x(time) power(1) generate(bs1)
*quadratic
bspline, x(time) power(2) generate(bs2)
*cubic
bspline, x(time) power(3) generate(bs3)


*analysis primary outcomes

*1 perinatal mortality*

*linear spline
logistic perimort bs1* ban1 ban2 i.mob i.pe i.ethn i.sex i.cs ///
            b2.ses b3.macat i.parity i.urban, noconstant
estat ic
*quadratic spline
logistic perimort bs2* ban1 ban2 i.mob i.pe i.ethn i.sex i.cs ///
b2.ses b3.macat i.parity i.urban, noconstant
estat ic
```

```
*cubic spline
logistic perimort bs3* ban1 ban2 i.mob i.pe i.ethn i.sex i.cs ///
        b2.ses b3.macat i.parity i.urban, noconstant
estat ic

*linear spline is best model based on AIC/BIC


*2 preterm birth*

*linear spline
logistic preterm bs1* ban1 ban2 i.mob i.pe i.ethn i.sex i.cs ///
        b2.ses b3.macat i.parity i.urban, noconstant
estat ic
*quadratic spline
logistic preterm bs2* ban1 ban2 i.mob i.pe i.ethn i.sex i.cs ///
        b2.ses b3.macat i.parity i.urban, noconstant
estat ic
*cubic spline - best model based on AIC/BIC
logistic preterm bs3* ban1 ban2 i.mob i.pe i.ethn i.sex i.cs ///
        b2.ses b3.macat i.parity i.urban, noconstant
estat ic

*cubic spline is best model based on AIC/BIC

*3 sga*

*linear spline
logistic sga bs1* ban1 ban2 i.mob i.pe i.ethn i.sex i.cs ///
        b2.ses b3.macat i.parity i.urban, noconstant
estat ic
*quadratic spline
logistic sga bs2* ban1 ban2 i.mob i.pe i.ethn i.sex i.cs ///
        b2.ses b3.macat i.parity i.urban, noconstant
estat ic
*cubic spline
logistic sga bs3* ban1 ban2 i.mob i.pe i.ethn i.sex i.cs ///
        b2.ses b3.macat i.parity i.urban, noconstant
estat ic

*linear spline is best model based on AIC/BIC

log close
```

```
***************************************
* MAIN ANALYSES OF SECONDARY OUTCOMES *
***************************************

set more off
log using "analyses-secondaryoutcomes.smcl", replace
use "masterfile.dta", clear

*drop chromosomal anomalies
drop if chroman == 1

*generate primary outcome variables

*stillbirth
generate sb = 0
replace sb = 1 if mortality == 0
replace sb = . if mortality == .

*early neonatal mortality
generate earlymort = 0
replace earlymort = 1 if mortality == 1
	*exclude cases of stillbirth or missing info on mortality status
	replace earlymort = . if mortality == 0
	replace earlymort = . if mortality == .

*very preterm birth
generate vpreterm = 0
replace vpreterm = 1 if gacat == 1
replace vpreterm = 1 if gacat == 2
	*exclude cases of stillbirth or missing info on mortality status
	replace vpreterm = . if mortality == 0
	replace vpreterm = . if mortality == .

*low birth weight
generate lbw = 0
replace lbw = 1 if bwcat == 1
replace lbw = 1 if bwcat == 2
	*exclude cases of stillbirth or missing info on mortality status
	replace lbw = . if mortality == 0
	replace lbw = . if mortality == .

*very low birth weight
generate vlbw = 0
replace vlbw = 1 if bwcat == 1
	*exclude cases of stillbirth or missing info on mortality status
	replace vlbw = . if mortality == 0
	replace vlbw = . if mortality == .
```

```
*very SGA: exclude cases of stillbirth or missing info on mortality status
replace vsga = . if mortality == 0
replace vsga = . if mortality == .

*re-categorise mode of delivery from categorical into dichotomous
*           (caesarean section yes/no)
generate cs = 0
replace cs = 1 if mod == 3
replace cs = 1 if mod == 4
replace cs = . if mod == .

*generate continuous time variable in months
generate time = (year-2000)*12 + mob

*generate ban dummy to model 2004 ban
generate ban1 = 0
replace ban1 = 1 if time>47
label variable ban1 "2004 ban"

*generate ban dummy to model 2008 ban
generate ban2 = 0
replace ban2 = 1 if time>101
label variable ban2 "2008 ban"

*generate bsplines to model non-linear time trends
*linear
bspline, x(time) power(1) generate(bs1)
*quadratic
bspline, x(time) power(2) generate(bs2)
*cubic
bspline, x(time) power(3) generate(bs3)

*analysis secondary outcomes

*1 stillbirth*

*linear spline
logistic sb bs1* ban1 ban2 i.mob i.pe i.ethn i.sex ///
            b2.ses b3.macat i.parity i.urban, noconstant
estat ic
*quadratic spline
logistic sb bs2* ban1 ban2 i.mob i.pe i.ethn i.sex ///
            b2.ses b3.macat i.parity i.urban, noconstant
estat ic
*cubic spline
logistic sb bs3* ban1 ban2 i.mob i.pe i.ethn i.sex ///
            b2.ses b3.macat i.parity i.urban, noconstant
estat ic
```

```
*linear spline is best model based on AIC/BIC

*2 early neonatal mortality*

*linear spline
logistic earlymort bs1* ban1 ban2 i.mob i.pe i.ethn i.sex i.cs ///
	b2.ses b3.macat i.parity i.urban, noconstant
estat ic
*quadratic spline
logistic earlymort bs2* ban1 ban2 i.mob i.pe i.ethn i.sex i.cs ///
	b2.ses b3.macat i.parity i.urban, noconstant
estat ic
*cubic spline
logistic earlymort bs3* ban1 ban2 i.mob i.pe i.ethn i.sex i.cs ///
	b2.ses b3.macat i.parity i.urban, noconstant
estat ic

*linear spline is best model based on AIC/BIC

*3 very preterm birth*

*linear spline
logistic vpreterm bs1* ban1 ban2 i.mob i.pe i.ethn i.sex i.cs ///
	b2.ses b3.macat i.parity i.urban, noconstant
estat ic
*quadratic spline
logistic vpreterm bs2* ban1 ban2 i.mob i.pe i.ethn i.sex i.cs ///
	b2.ses b3.macat i.parity i.urban, noconstant
estat ic
*cubic spline
logistic vpreterm bs3* ban1 ban2 i.mob i.pe i.ethn i.sex i.cs ///
	b2.ses b3.macat i.parity i.urban, noconstant
estat ic

*cubic spline is best model based on AIC/BIC

*4 low birth weight*

*linear spline
logistic lbw bs1* ban1 ban2 i.mob i.pe i.ethn i.sex i.cs ///
	b2.ses b3.macat i.parity i.urban, noconstant
estat ic
*quadratic spline
logistic lbw bs2* ban1 ban2 i.mob i.pe i.ethn i.sex i.cs ///
	b2.ses b3.macat i.parity i.urban, noconstant
estat ic
*cubic spline
```

```
logistic lbw bs3* ban1 ban2 i.mob i.pe i.ethn i.sex i.cs ///
	b2.ses b3.macat i.parity i.urban, noconstant
estat ic

*cubic spline is best model based on AIC/BIC

*5 very low birth weight*

*linear spline
logistic vlbw bs1* ban1 ban2 i.mob i.pe i.ethn i.sex i.cs ///
	b2.ses b3.macat i.parity i.urban, noconstant
estat ic
*quadratic spline
logistic vlbw bs2* ban1 ban2 i.mob i.pe i.ethn i.sex i.cs ///
	b2.ses b3.macat i.parity i.urban, noconstant
estat ic
*cubic spline
logistic vlbw bs3* ban1 ban2 i.mob i.pe i.ethn i.sex i.cs ///
	b2.ses b3.macat i.parity i.urban, noconstant
estat ic

*cubic spline is best model based on AIC/BIC

*6 very sga*

*linear spline
logistic vsga bs1* ban1 ban2 i.mob i.pe i.ethn i.sex i.cs ///
	b2.ses b3.macat i.parity i.urban, noconstant
estat ic
*quadratic spline
logistic vsga bs2* ban1 ban2 i.mob i.pe i.ethn i.sex i.cs ///
	b2.ses b3.macat i.parity i.urban, noconstant
estat ic
*cubic spline
logistic vsga bs3* ban1 ban2 i.mob i.pe i.ethn i.sex i.cs ///
	b2.ses b3.macat i.parity i.urban, noconstant
estat ic

*linear spline is best model based on AIC/BIC

*7 congenital anomalies*

*linear spline
logistic congan bs1* ban1 ban2 i.mob i.pe i.ethn i.sex ///
	b2.ses b3.macat i.parity i.urban, noconstant
estat ic
*quadratic spline
logistic congan bs2* ban1 ban2 i.mob i.pe i.ethn i.sex ///
```

```
        b2.ses b3.macat i.parity i.urban, noconstant
estat ic
*cubic spline
logistic congan bs3* ban1 ban2 i.mob i.pe i.ethn i.sex ///
        b2.ses b3.macat i.parity i.urban, noconstant
estat ic

*quadratic spline is best model based on AIC/BIC

log close


*******************************************************************************
* ANALYSES OF PRIMARY OUTCOMES WITH MULTIPLE IMPUTATION OF MISSING COVARIATES *
*******************************************************************************

*1 perinatal mortality*

set more off
log using "analyses-primary outcomes-MI-perinatal mortality.smcl", replace
use "masterfile.dta", clear

*drop chromosomal anomalies
drop if chroman == 1

*generate primary outcome variable

*perinatal mortality
generate perimort = 0
replace perimort = 1 if mortality == 1
        *exclude cases of stillbirth or missing info on mortality status
        replace perimort = 1 if mortality == 0
        replace perimort = . if mortality == .

*re-categorise mode of delivery from categorical into dichotomous
*       (caesarean section yes/no)
generate cs = 0
replace cs = 1 if mod == 3
replace cs = 1 if mod == 4
replace cs = . if mod == .

*generate continuous time variable in months
generate time = (year-2000)*12 + mob

*generate ban dummy to model 2004 ban
generate ban1 = 0
replace ban1 = 1 if time>47
label variable ban1 "2004 ban"
```

```
*generate ban dummy to model 2008 ban
generate ban2 = 0
replace ban2 = 1 if time>101
label variable ban2 "2008 ban"

*generate bsplines to model time trend
*linear
bspline, x(time) power(1) generate(bs1)

*set dataset for MI
mi set wide

*define which variables need imputing
mi register imputed macat ethn ses urban parity pe cs sex

*explore patterns of missingness
mi misstable sum

*impute variables
mi impute chained (ologit) macat ethn ses urban parity pe cs sex = ga bw ///
        perimort, add(5) force rseed(25831)

*run optimal model as identified in primary analysis
mi estimate: logistic perimort bs1* ban1 ban2 i.mob i.pe i.ethn i.sex i.cs ///
        b2.ses b3.macat i.parity i.urban, noconstant

log close

*2 preterm birth*

set more off
log using "analyses-primary outcomes-MI-preterm birth.smcl", replace
use "masterfile.dta", clear
```

```
*drop chromosomal anomalies
drop if chroman == 1

*generate primary outcome variable

*preterm birth: exclude cases of stillbirth or missing info on mortality status
replace preterm = . if mortality == 0
replace preterm = . if mortality == .

*re-categorise mode of delivery from categorical into dichotomous
*        (caesarean section yes/no)
generate cs = 0
replace cs = 1 if mod == 3
replace cs = 1 if mod == 4
replace cs = . if mod == .

*generate continuous time variable in months
generate time = (year-2000)*12 + mob

*generate ban dummy to model 2004 ban
generate ban1 = 0
replace ban1 = 1 if time>47
label variable ban1 "2004 ban"

*generate ban dummy to model 2008 ban
generate ban2 = 0
replace ban2 = 1 if time>101
label variable ban2 "2008 ban"

*generate bsplines to model non-linear time trends
*cubic
bspline, x(time) power(3) generate(bs3)

*set dataset for MI
mi set wide

*define which variables need imputing
mi register imputed macat ethn ses urban parity pe cs sex

*explore patterns of missingness
mi misstable sum

*impute variables
mi impute chained (ologit) macat ethn ses urban parity pe cs sex = ga bw ///
        preterm, add(5) force rseed(25831)

*run optimal model as identified in primary analysis
mi estimate: logistic preterm bs3* ban1 ban2 i.mob i.pe i.ethn i.sex i.cs ///
```

```
	b2.ses b3.macat i.parity i.urban, noconstant

log close

*3 SGA*

set more off
log using "analyses-primary outcomes-MI-SGA.smcl", replace
use "masterfile.dta", clear

*drop chromosomal anomalies
drop if chroman == 1

*generate primary outcome variables

*SGA: exclude cases of stillbirth or missing info on mortality status
replace sga = . if mortality == 0
replace sga = . if mortality == .

*re-categorise mode of delivery from categorical into dichotomous
*	(caesarean section yes/no)
generate cs = 0
replace cs = 1 if mod == 3
replace cs = 1 if mod == 4
replace cs = . if mod == .

*generate continuous time variable in months
generate time = (year-2000)*12 + mob

*generate ban dummy to model 2004 ban
generate ban1 = 0
replace ban1 = 1 if time>47
label variable ban1 "2004 ban"

*generate ban dummy to model 2008 ban
generate ban2 = 0
replace ban2 = 1 if time>101
label variable ban2 "2008 ban"

*generate bsplines to model time trend
bspline, x(time) power(1) generate(bs1)

*set dataset for MI
mi set wide

*define which variables need imputing
mi register imputed macat ethn ses urban parity pe cs sex
```

```
*explore patterns of missingness
mi misstable sum

*impute variables
mi impute chained (ologit) macat ethn ses urban parity pe cs sex = ga bw ///
        sga, add(5) force rseed(25831)

*run optimal model as identified in primary analysis
mi estimate: logistic sga bs1* ban1 ban2 i.mob i.pe i.ethn i.sex i.cs ///
        b2.ses b3.macat i.parity i.urban, noconstant

log close


*******************************************
* SENSITIVITY ANALYSES OF PRIMARY OUTCOMES *
*******************************************

set more off
log using "analyses-primary outcomes-sensitivity.smcl", replace
use "masterfile.dta", clear

*drop chromosomal anomalies
drop if chroman == 1

*generate primary outcome variables

*preterm birth: exclude cases of stillbirth or missing info on mortality status
replace preterm = . if mortality == 0
replace preterm = . if mortality == .

*SGA: exclude cases of stillbirth or missing info on mortality status
replace sga = . if mortality == 0
replace sga = . if mortality == .

*re-categorise mode of delivery from categorical into dichotomous
*       (caesarean section yes/no)
generate cs = 0
replace cs = 1 if mod == 3
replace cs = 1 if mod == 4
replace cs = . if mod == .

*generate continuous time variable in months
generate time = (year-2000)*12 + mob

*generate ban dummy to model 2004 ban
generate ban1 = 0
replace ban1 = 1 if time>47
```

```
label variable ban1 "2004 ban"

*generate ban dummy to model 2008 ban
generate ban2 = 0
replace ban2 = 1 if time>101
label variable ban2 "2008 ban"

*generate bsplines to model (non-linear) time trends
*linear
bspline, x(time) power(1) generate(bs1)
*quadratic
bspline, x(time) power(2) generate(bs2)
*cubic
bspline, x(time) power(3) generate(bs3)

*1 preterm birth indicated vs. spontaneous*

*1a indicated preterm birth

*generate indicated preterm birth indicator
generate pretermind = 0
replace pretermind = 1 if preterm==1 & induced==1
replace pretermind = . if mortality ==.
replace pretermind = . if mortality ==0
replace pretermind = . if preterm==1 & induced==0
replace pretermind = . if preterm==1 & induced==.

*linear spline
logistic preterm bs1* ban1 ban2 i.mob i.pe i.ethn i.sex i.cs ///
        b2.ses b3.macat i.parity i.urban, noconstant
estat ic
*quadratic spline
logistic preterm bs2* ban1 ban2 i.mob i.pe i.ethn i.sex i.cs ///
        b2.ses b3.macat i.parity i.urban, noconstant
estat ic
*cubic spline
logistic pretermind bs3* ban1 ban2 i.mob i.pe i.ethn i.sex i.cs ///
        b2.ses b3.macat i.parity i.urban, noconstant
estat ic

*cubic spline is best model based on AIC/BIC

*1b spontaneous preterm birth*

*generate spontaneous preterm birth indicator
generate pretermsp = 0
replace pretermsp = 1 if preterm==1 & induced==0
replace pretermsp = . if mortality ==.
```

```
replace pretermsp = . if mortality ==0
replace pretermsp = . if preterm==1 & induced==1
replace pretermsp = . if preterm==1 & induced==.

*linear spline
logistic preterm bs1* ban1 ban2 i.mob i.pe i.ethn i.sex i.cs ///
	b2.ses b3.macat i.parity i.urban, noconstant
estat ic
*quadratic spline
logistic preterm bs2* ban1 ban2 i.mob i.pe i.ethn i.sex i.cs ///
	b2.ses b3.macat i.parity i.urban, noconstant
estat ic
*cubic spline
logistic pretermsp bs3* ban1 ban2 i.mob i.pe i.ethn i.sex i.cs ///
	b2.ses b3.macat i.parity i.urban, noconstant
estat ic

*cubic spline is best model based on AIC/BIC

*2 analyses restricted to cases where gestational age recorded as 'certain'*

*note: certainty of GA was not an item in 2000 and 2001 datasets
*	so analysis restricted to 2002-2011
*	comparison therefore with complete case analysis of 2002-2011 for pretem birth

*keep only years 2002-2011:
drop if year == 2000
drop if year == 2001

*preterm birth: define model based on full population 2002-2011:
*linear spline
logistic preterm bs1* ban1 ban2 i.mob i.pe i.ethn i.sex i.cs ///
	b2.ses b3.macat i.parity i.urban, noconstant
estat ic
*quadratic spline
logistic preterm bs2* ban1 ban2 i.mob i.pe i.ethn i.sex i.cs ///
	b2.ses b3.macat i.parity i.urban, noconstant
estat ic
*cubic spline - best model based on AIC/BIC
logistic preterm bs3* ban1 ban2 i.mob i.pe i.ethn i.sex i.cs ///
	b2.ses b3.macat i.parity i.urban, noconstant
estat ic

*cubic spline is best model based on AIC/BIC
l
*sga: define model based on full population 2002-2011:
*linear spline
logistic sga bs1* ban1 ban2 i.mob i.pe i.ethn i.sex i.cs ///
```

```
            b2.ses b3.macat i.parity i.urban, noconstant
estat ic
*quadratic spline
logistic sga bs2* ban1 ban2 i.mob i.pe i.ethn i.sex i.cs ///
            b2.ses b3.macat i.parity i.urban, noconstant
estat ic
*cubic spline
logistic sga bs3* ban1 ban2 i.mob i.pe i.ethn i.sex i.cs ///
            b2.ses b3.macat i.parity i.urban, noconstant
estat ic

*linear spline is best model based on AIC/BIC

*2a preterm birth: only cases with 'certain' gestational age*

*generate indicator for gestational age = 'certain'
generate gacertain = 0
replace gacertain = . if mortality == 0
replace gacertain = 1 if g_zek == 1
replace gacertain = 1 if v_zek == 1

*keep only cases with 'certain' gestational age
keep if gacertain == 1

*cubic spline
logistic preterm bs3* ban1 ban2 i.mob i.pe i.ethn i.sex i.cs ///
            b2.ses b3.macat i.parity i.urban, noconstant

*2a SGA: only cases with 'certain' gestational age*

*linear spline - best model based on AIC/BIC
logistic sga bs1* ban1 ban2 i.mob i.pe i.ethn i.sex i.cs ///
            b2.ses b3.macat i.parity i.urban, noconstant
```

```
*3 sensitivity analysis restricted to cases born at >=26wks*

use "masterfile.dta", clear

*drop chromosomal anomalies
drop if chroman == 1

*generate primary outcome variables

*perinatal mortality
generate perimort = 0
replace perimort = 1 if mortality == 1
        *exclude cases of stillbirth or missing info on mortality status
        replace perimort = 1 if mortality == 0
        replace perimort = . if mortality == .

*preterm birth: exclude cases of stillbirth or missing info on mortality status
replace preterm = . if mortality == 0
replace preterm = . if mortality == .

*SGA: exclude cases of stillbirth or missing info on mortality status
replace sga = . if mortality == 0
replace sga = . if mortality == .

*re-categorise mode of delivery from categorical into dichotomous
*       (caesarean section yes/no)
generate cs = 0
replace cs = 1 if mod == 3
replace cs = 1 if mod == 4
replace cs = . if mod == .

*generate continuous time variable in months
generate time = (year-2000)*12 + mob

*generate ban dummy to model 2004 ban
generate ban1 = 0
replace ban1 = 1 if time>47
label variable ban1 "2004 ban"

*generate ban dummy to model 2008 ban
generate ban2 = 0
replace ban2 = 1 if time>101
label variable ban2 "2008 ban"

*generate bsplines to model (non-linear) time trends
*linear
bspline, x(time) power(1) generate(bs1)
*quadratic
```

```
bspline, x(time) power(2) generate(bs2)
*cubic
bspline, x(time) power(3) generate(bs3)

*keep only cases born at gestational age >=26 weeks
drop if ga<26

*3a preterm birth: restricted to cases born at >=26wks
*cubic spline - best model based on AIC/BIC
logistic preterm bs3* ban1 ban2 i.mob i.pe i.ethn i.sex i.cs ///
        b2.ses b3.macat i.parity i.urban, noconstant

*3b perinatal mortality: restricted to cases born at >=26wks
*linear spline - best model based on AIC/BIC
logistic perimort bs1* ban1 ban2 i.mob i.pe i.ethn i.sex i.cs ///
        b2.ses b3.macat i.parity i.urban, noconstant

log close


*************************
* COUNTERFACTUAL MODELS *
*************************

set more off
log using "analyses-counterfactual.smcl", replace
use "masterfile.dta", clear

*1 SGA: counterfactual estimates

*drop chromosomal anomalies
drop if chroman == 1

*generate primary outcome variables
*SGA
replace sga = . if mortality == 0
replace sga = . if mortality == .

*re-categorise confounders where necessary
generate cs = 0
replace cs = 1 if mod == 3
replace cs = 1 if mod == 4
replace cs = . if mod == .

*generate continuous time variable in months
generate time = (year-2000)*12 + mob

*generate ban dummy to model 2004 ban
```

```
generate ban1 = 0
replace ban1 = 1 if time>47
label variable ban1 "2004 ban"

*generate ban dummy to model 2008 ban
generate ban2 = 0
replace ban2 = 1 if time>101
label variable ban2 "2008 ban"

*generate bsplines to model non-linear time trends
*linear
bspline, x(time) power(1) generate(bs1)

*linear spline - best model based on AIC/BIC
logit sga bs1* ban1 ban2 i.mob i.pe i.ethn i.sex i.cs ///
	b2.ses b3.macat i.parity i.urban, noconstant

*generate dummy variables for categorical variables for calculating
*	counterfactual rates

*month
generate feb=0
replace feb=1 if mob==2
generate mar=0
replace mar=1 if mob==3
generate apr=0
replace apr=1 if mob==4
generate may=0
replace may=1 if mob==5
generate jun=0
replace jun=1 if mob==6
generate jul=0
replace jul=1 if mob==7
generate aug=0
replace aug=1 if mob==8
generate sep=0
replace sep=1 if mob==9
generate oct=0
replace oct=1 if mob==10
generate nov=0
replace nov=1 if mob==11
generate dec=0
replace dec=1 if mob==12

*ethnicity
generate eth2=0
replace eth2=1 if ethn==2
generate eth3=0
```

```
replace eth3=1 if ethn==3
generate eth4=0
replace eth4=1 if ethn==4
generate eth5=0
replace eth5=1 if ethn==5

*SES
generate ses1=0
replace ses1=1 if ses==1
generate ses3=0
replace ses3=1 if ses==3

*maternal age
generate macat1=0
replace macat1=1 if macat==1
generate macat2=0
replace macat2=1 if macat==2
generate macat4=0
replace macat4=1 if macat==4
generate macat5=0
replace macat5=1 if macat==5
generate macat6=0
replace macat6=1 if macat==6

*sex
generate female=0
replace female=1 if sex==2

*calculate counterfactual estimates for individual cases based on Beta values
*       derived from primary model, leaving out 2008 smoking ban indicator
generate sgapr1 = -2.33471*bs11-2.562213*bs12-.014287*ban1-.0196977*feb-.021213* ///
        mar-.0406058*apr-.0397418*may-.0351222*jun-.0366112*jul-.0037197*aug-.0395109* ///
        sep-.024721*oct-.0386926*nov+.0005786*dec+.8044962*pe+.1049532*eth2+.5559078* ///
        eth3+.1893123*eth4+.3349467*eth5+.0153442*female+.1789847*cs-.0921885*ses1 ///
        +.1564516*ses3+.3126169*macat1+.1898468*macat2-.0636316*macat4+.0343234* ///
        macat5+.1832075*macat6+.0466379*parity+.0340967*urban
generate sgapr2 = exp(-sgapr1)
generate sgapr3 = 1/(1+sgapr2)
```

```
*keep only cases with complete data on all covariates
generate count = 1
sort time
generate complete = 1
replace complete = 0 if mob==.
replace complete = 0 if pe==.
replace complete = 0 if ethn==.
replace complete = 0 if sex==.
replace complete = 0 if cs==.
replace complete = 0 if ses==.
replace complete = 0 if macat==.
replace complete = 0 if parity==.
replace complete = 0 if urban==.
replace sga = . if complete == 0
replace sgapr3 = . if complete == 0

*calculate difference between actual occurrence of SGA and counterfactual estimates
generate sgaprev = sgapr3-sga

*restrict analyses to post 2008 ban period
drop if ban2==0

*calculate sum of individual differences between actual occurrence of SGA and
*         counterfactual estimates
egen sgaprevtot = sum(sgaprev)
summarize sgaprevtot


*2 very preterm birth: counterfactual estimates
use "masterfile.dta", clear

*drop chromosomal anomalies
drop if chroman == 1

*generate primary outcome variables
generate vptb = 0
replace vptb = 1 if gacat == 1
replace vptb = 1 if gacat == 2
replace vptb = . if mortality == 0
replace vptb = . if mortality == .

*re-categorise confounders where necessary
generate cs = 0
replace cs = 1 if mod == 3
replace cs = 1 if mod == 4
replace cs = . if mod == .

*generate continuous time variable in months
```

```
generate time = (year-2000)*12 + mob

*generate ban dummy to model 2004 ban
generate ban1 = 0
replace ban1 = 1 if time>47
label variable ban1 "2004 ban"

*generate ban dummy to model 2008 ban
generate ban2 = 0
replace ban2 = 1 if time>101
label variable ban2 "2008 ban"

*generate bsplines to model non-linear time trends
*cubic
bspline, x(time) power(3) generate(bs3)

*cubic spline - best model based on AIC/BIC
logit vptb bs3* ban1 ban2 i.mob i.pe i.ethn i.sex i.cs ///
        b2.ses b3.macat i.parity i.urban, noconstant

*generate dummy variables for categorical variables for calculating
*       counterfactual rates

*month
generate feb=0
replace feb=1 if mob==2
generate mar=0
replace mar=1 if mob==3
generate apr=0
replace apr=1 if mob==4
generate may=0
replace may=1 if mob==5
generate jun=0
replace jun=1 if mob==6
generate jul=0
replace jul=1 if mob==7
generate aug=0
replace aug=1 if mob==8
generate sep=0
replace sep=1 if mob==9
generate oct=0
replace oct=1 if mob==10
generate nov=0
replace nov=1 if mob==11
generate dec=0
replace dec=1 if mob==12

*ethnicity
```

```
generate eth2=0
replace eth2=1 if ethn==2
generate eth3=0
replace eth3=1 if ethn==3
generate eth4=0
replace eth4=1 if ethn==4
generate eth5=0
replace eth5=1 if ethn==5

*SES
generate ses1=0
replace ses1=1 if ses==1
generate ses3=0
replace ses3=1 if ses==3

*maternal age
generate macat1=0
replace macat1=1 if macat==1
generate macat2=0
replace macat2=1 if macat==2
generate macat4=0
replace macat4=1 if macat==4
generate macat5=0
replace macat5=1 if macat==5
generate macat6=0
replace macat6=1 if macat==6

*sex
generate female=0
replace female=1 if sex==2

*calculate counterfactual estimates for individual cases based on Beta values
*         derived from primary model, leaving out 2008 smoking ban indicator
generate vptbpr1 = -1.865873*bs31-6.327686*bs32-4.597792*bs33-7.315962*bs34-.0607096*ban1-
.0408812*feb-.0300124* ///
         mar+.0331726*apr+.0546653*may+.1477543*jun+.0487273*jul+.0099162*aug-.0338381* ///
         sep+.048534*oct+.0772596*nov+.0401041*dec+2.025254*pe+.0216269*eth2+.4900425* ///
         eth3-.0011279*eth4+.2680504*eth5-.1819819*female+1.501927*cs-.0840324*ses1 ///
         +.1357444*ses3+.6320986*macat1+.2143648*macat2-.0989038*macat4-.0836981* ///
         macat5+.121771*macat6-.3169793*parity+.0351782*urban
generate vptbpr2 = exp(-vptbpr1)
generate vptbpr3 = 1/(1+vptbpr2)

*keep only cases with complete data on all covariates
generate count = 1
sort time
generate complete = 1
replace complete = 0 if mob==.
```

```
replace complete = 0 if pe==.
replace complete = 0 if ethn==.
replace complete = 0 if sex==.
replace complete = 0 if cs==.
replace complete = 0 if ses==.
replace complete = 0 if macat==.
replace complete = 0 if parity==.
replace complete = 0 if urban==.
replace vptb = . if complete == 0
replace vptbpr3 = . if complete == 0

*calculate difference between actual occurrence of very preterm birth
* and counterfactual estimates
generate vptbprev = vptbpr3-vptb

*restrict analyses to post 2008 ban period
drop if ban2==0

*calculate sum of individual differences between actual occurrence of very
*           preterm birth and counterfactual estimates
egen vptbprevtot = sum(vptbprev)
summarize vptbprevtot

*3 very SGA: counterfactual estimates
use "masterfile.dta", clear

*drop chromosomal anomalies
drop if chroman == 1

*generate secondary outcome variables
*vSGA
replace vsga = . if mortality == 0
replace vsga = . if mortality == .

*re-categorise confounders where necessary
generate cs = 0
replace cs = 1 if mod == 3
replace cs = 1 if mod == 4
replace cs = . if mod == .

*generate continuous time variable in months
generate time = (year-2000)*12 + mob

*generate ban dummy to model 2004 ban
generate ban1 = 0
replace ban1 = 1 if time>47
label variable ban1 "2004 ban"
```

```
*generate ban dummy to model 2008 ban
generate ban2 = 0
replace ban2 = 1 if time>101
label variable ban2 "2008 ban"

*generate bsplines to model non-linear time trends
*linear
bspline, x(time) power(1) generate(bs1)

*vsga
*linear spline - best model based on AIC/BIC
logit vsga bs1* ban1 ban2 i.mob i.pe i.ethn i.sex i.cs ///
	b2.ses b3.macat i.parity i.urban, noconstant

*generate dummy variables for categorical variables for calculating
*	counterfactual rates

*month
generate feb=0
replace feb=1 if mob==2
generate mar=0
replace mar=1 if mob==3
generate apr=0
replace apr=1 if mob==4
generate may=0
replace may=1 if mob==5
generate jun=0
replace jun=1 if mob==6
generate jul=0
replace jul=1 if mob==7
generate aug=0
replace aug=1 if mob==8
generate sep=0
replace sep=1 if mob==9
generate oct=0
replace oct=1 if mob==10
generate nov=0
replace nov=1 if mob==11
generate dec=0
replace dec=1 if mob==12

*ethnicity
generate eth2=0
replace eth2=1 if ethn==2
generate eth3=0
replace eth3=1 if ethn==3
generate eth4=0
replace eth4=1 if ethn==4
```

```
generate eth5=0
replace eth5=1 if ethn==5

*SES
generate ses1=0
replace ses1=1 if ses==1
generate ses3=0
replace ses3=1 if ses==3

*maternal age
generate macat1=0
replace macat1=1 if macat==1
generate macat2=0
replace macat2=1 if macat==2
generate macat4=0
replace macat4=1 if macat==4
generate macat5=0
replace macat5=1 if macat==5
generate macat6=0
replace macat6=1 if macat==6

*sex
generate female=0
replace female=1 if sex==2

*calculate counterfactual estimates for individual cases based on Beta values
*	derived from primary model, leaving out 2008 smoking ban indicator
generate vsgapr1 = -3.765832*bs11-4.259749*bs12+.0217235*ban1-.0265496*feb-.0603582* ///
	mar-.0691322*apr-.0777018*may-.0509605*jun-.0352004*jul-.01131*aug-.0242428* ///
	sep-.0545836*oct-.0485384*nov+.0134524*dec+.7745957*pe+.0273653*eth2+.4899592* ///
	eth3+.1015255*eth4+.3202685*eth5+.0131471*female+.5587565*cs-.1456107*ses1 ///
	+.2207008*ses3+.3162824*macat1+.2144919*macat2-.0684586*macat4+.0781254* ///
	macat5+.3082961*macat6+.0312618*parity+.0072711*urban
generate vsgapr2 = exp(-vsgapr1)
generate vsgapr3 = 1/(1+vsgapr2)

*keep only cases with complete data on all covariates
generate count = 1
sort time
generate complete = 1
replace complete = 0 if mob==.
replace complete = 0 if pe==.
replace complete = 0 if ethn==.
replace complete = 0 if sex==.
replace complete = 0 if cs==.
replace complete = 0 if ses==.
replace complete = 0 if macat==.
replace complete = 0 if parity==.
```

```
replace complete = 0 if urban==.
replace vsga = . if complete == 0
replace vsgapr3 = . if complete == 0

*calculate difference between actual occurrence of very SGA and counterfactual estimates
generate vsgaprev = vsgapr3-vsga

*restrict analyses to post 2008 ban period
drop if ban2==0

*calculate sum of individual differences between actual occurrence of very SGA and
*           counterfactual estimates
egen vsgaprevtot = sum(vsgaprev)
summarize vsgaprevtot

log close
```
